# Trends and Key Factors Associated With Racial and Ethnic Differences in Life’s Essential 8 Scores

**DOI:** 10.1001/jamanetworkopen.2025.16663

**Published:** 2025-06-18

**Authors:** Huanhuan Yang, Chenxi Huang, Mitsuaki Sawano, Jeph Herrin, Kamil F. Faridi, Zhihui Li, Erica Spatz, Harlan M. Krumholz, Yuan Lu

**Affiliations:** 1 Center for Outcomes Research and Evaluation, Yale New Haven Hospital, New Haven, Connecticut; 2 Vanke School of Public Health, Tsinghua University, Beijing, China; 3 Section of Cardiovascular Medicine, Department of Internal Medicine, Yale School of Medicine, New Haven, Connecticut; 4 Department of Health Policy and Management, Yale School of Public Health, New Haven, Connecticut; 5 Department of Biomedical Informatics and Data Science, Yale School of Medicine, New Haven, Connecticut; 6 Department of Epidemiology (Chronic Diseases), Yale School of Public Health, New Haven, Connecticut

## Abstract

**Importance:**

Significant racial and ethnic differences exist in Life’s Essential 8 (LE8), but the trends in these differences over time are not well understood. Additionally, the key components of LE8 associated with these differences are unclear.

**Objectives:**

To evaluate trends in racial and ethnic differences in LE8 over a 10-year period and to identify the primary factors associated with the LE8 differences.

**Design, Setting, and Participants:**

Serial population-based cross-sectional study of the National Health and Nutrition Examination Survey from 2011 to 2020 that included adults aged 20 to 79 years. The analysis was performed between March and October 2024.

**Exposure:**

Self-reported race and ethnicity.

**Main Outcome and Measures:**

Trends in racial and ethnic differences in LE8 and primary factors associated with the differences.

**Results:**

The median (IQR) age of the 16 104 participants was 46 (32-59) years; 8262 (51.1%) were women; 1974 (5.2%) were Asian, 3918 (10.9%) were Black, 4144 (15.7%) were Latino/Hispanic, and 6068 (68.2%) were White. From 2011 to 2020, Asian adults had the highest LE8 score (71.2; 95% CI, 70.3-72.0), followed by White (67.7; 95% CI, 66.9-68.6) and Latino/Hispanic (65.9; 95% CI, 61.3-62.7) adults, and Black adults (62.0) had the lowest LE8 score. These racial and ethnic differences in LE8 overall score did not significantly change from 2011 to 2020. However, the differences in several individual components of LE8 changed significantly. For example, the Latino/Hispanic vs White difference in sleep health score significantly increased, from −1.25 to −4.38, with a descriptive difference-of-differences of −3.12 (95% CI, −5.83 to −0.42; *P* = .02). In 2017 to 2020, all but blood lipids and nicotine exposure were negatively associated factors (*z* scores <0) for the Black vs White difference; nicotine exposure was the key positive (*z *score = 1.01), while physical activity was the key negative (*z* score = −1.01) factor associated with the Latino/Hispanic vs White difference; nicotine exposure (*z *score = 2.59) and diet (*z *score = 2.12) were the primary positive factors associated with Asian vs White difference.

**Conclusions and Relevance:**

In this cross-sectional study, racial and ethnic differences in overall LE8 scores compared with White adults remained largely unchanged from 2011 to 2020. These differences were associated with varying components across different racial and ethnic groups, emphasizing the need for targeted, group-specific interventions.

## Key Points

**Question:**

What are the trends in racial and ethnic differences in Life’s Essential 8 (LE8) metrics, and what are the primary factors associated with these differences over the past decade?

**Findings:**

In this cross-sectional study of 16 104 participants, overall racial and ethnic differences in LE8 scores remained relatively stable over the past decade, although disparities in individual components shifted. Differences between Black and White participants were primarily associated with factors other than blood lipids and nicotine exposure, Latino/Hispanic vs White differences were largely attributable to lower levels of physical activity, and Asian vs White differences were favorably associated with healthier diet and lower nicotine exposure.

**Meaning:**

These findings suggest the importance of tailored interventions and offer actionable insights to address the distinct cardiovascular health needs of different racial and ethnic groups.

## Teaser

This cross-sectional study evaluates trends in racial and ethnic differences in Life's Essential 8 (LE8) scores over the 10-year period from 2011 to 2020.

## Introduction

Significant racial and ethnic disparities persist in cardiovascular health (CVH), contributing to unequal burdens of cardiovascular diseases (CVD) across populations.^1,2^ CVD remains the leading cause of death in the US, with disproportionate impacts on racial and ethnic minority groups, such as Black, Hispanic, and Asian populations.^3,4,5^ Despite numerous public health efforts aimed at improving CVH, these disparities continue to drive inequities in health outcomes. As the American Heart Association (AHA) shifted from Life’s Simple 7 to the more comprehensive Life’s Essential 8 (LE8) framework in 2022, a broader approach to health promotion and disease prevention has emerged, highlighting the need to examine how well we are addressing these disparities.^6,7^

Despite this shift in focus, there is a significant gap in our understanding of how racial and ethnic disparities in LE8 scores have evolved over the past decade. Previous studies have highlighted persistent disparities in CVH and have evaluated overall trends using LE8 metrics. However, comprehensive assessments of national trends specifically regarding racial and ethnic disparities in LE8 scores remain limited.^8,9,10,11^ Additionally, the relative contributions of each component of LE8 remain unclear. Without understanding which factors drive these disparities, it is difficult to develop targeted interventions that can reduce the gaps and improve health equity.

Understanding the trends of racial and ethnic differences in LE8 scores is critically important, as addressing health disparities is a national priority. Various public health measures, such as guidelines, policy interventions, and community outreach programs, have been implemented to reduce these inequities.^12,13^ However, it remains unclear whether these efforts have had a meaningful impact on closing racial and ethnic gaps in CVH. Given the continued high burden of CVD in minority populations and the recent introduction of the LE8 framework, this is an opportune moment to investigate how these differences have changed and whether specific components of LE8 have improved or worsened over time. To address this gap, our study used data from the National Health and Nutrition Examination Survey (NHANES) from 2011 to 2020 to assess trends in racial and ethnic differences in LE8 metrics and the relative association between each LE8 component with these differences among US adults.

## Methods

### Study Design

The NHANES is a nationally representative cross-sectional study conducted by the US Centers for Disease Control and Prevention (CDC) that collects health and nutrition data from a sample of the civilian, noninstitutionalized US population.^14^ We analyzed 2011 to 2020 data (4 cycles) combining household interviews and subsequent examinations at a mobile examination center (MEC), including anthropometric measurements and laboratory blood tests. The National Center for Health Statistics research ethics review board approved all survey protocols, and written informed consent was obtained from all participants. Detailed methodology is available from the CDC.^15^ This manuscript followed the Strengthening the Reporting of Observational Studies in Epidemiology (STROBE) reporting guideline.

### Study Participants

From 2011 to 2020, the continuous NHANES survey included a total of 45 462 participants. We restricted our analysis to 23 570 adults aged 20 to 79 years who self-identified as non-Hispanic Asian, non-Hispanic Black, Latino/Hispanic, or non-Hispanic White, excluding participants from other racial and ethnic categories (eg, other, unknown, or multiple; 937 participants). Further exclusions included those pregnant or lactating (425 participants) and individuals reporting a history of coronary heart disease (CHD), angina, heart attack, or stroke (1977 participants), resulting in 21 168 participants. For analyses of individual LE8 components, participants missing data for the specific component under study were excluded, maximizing available sample sizes. For instance, diet score analyses included 18 303 participants after excluding those with missing diet information (2867 participants). In contrast, for the analysis of overall LE8 score, we excluded participants with any missing component data (5064 participants), resulting in a final sample size of 16 104 participants (eFigure 1 in Supplement 1).

### LE8 Calculation and Covariates

The LE8 score (0-100), derived from the AHA’s methods for quantifying CVH, encompasses 8 components: diet, physical activity, nicotine exposure, sleep health, body mass index (BMI), blood glucose, blood lipids, and blood pressure, each rated on a scale from 0 to 100.^7^ Diet was assessed via a Dietary Approaches to Stop Hypertension (DASH)-style score using 24-hour recall data (8 food/nutrient groups; maximum 40 points).^16^ Health behaviors (physical activity, nicotine exposure, and sleep) were assessed via structured interviews, while clinical measures (BMI and blood pressure) and laboratory indicators (blood glucose and lipids) were obtained through standardized clinical examinations. The overall LE8 score was categorized as low (<50), medium (50-79), or high (≥80) CVH.^7^

Demographic and socioeconomic covariates included race and ethnicity (Asian, Black, Latino/Hispanic, and White), age, sex, the ratio of family income to poverty, education, marital status, and insurance. Medication use (hypertension, diabetes, hyperlipidemia) and depression (Patient Health Questionnaire [PHQ-2] score ≥3)^17^ were self-reported. Full scoring details, variable definitions, and categorization thresholds are provided in eMethods and eTable 1 in Supplement 1.

### Statistical Analysis

We accounted for NHANES’s complex sampling design using appropriate weights: MEC weights for the composite LE8 score, dietary day-1 weights for DASH diet, interview weights for physical activity, nicotine exposure, and sleep health, and MEC weights for clinical measures (BP, BMI, blood glucose, and blood lipids). Age standardization was performed using the direct standardization method with 5-year intervals based on the 2020 US Census population.^18^

Total and component LE8 scores were stratified by race and ethnicity and survey year. Racial and ethnic differences were quantified by subtracting White adults’ LE8 scores from Asian, Black, and Latino/Hispanic groups’ scores, tested via *z* tests. The temporal trends were assessed using survey-weighted regression models with year-race interaction terms, and showed as a descriptive difference-of-differences (dDoD). Unlike classical difference-in-differences methods, this descriptive approach does not imply any causal interpretations.

To evaluate the relative association for each component, we calculated *z* scores for the differences with White adults across all components to account for differences in scale. *z* Scores with positive or negative values were considered positive or negative factors, respectively. To evaluate the relative magnitude of race and ethnicity difference in each component, we calculated *z* scores for the differences with White adults across all components to account for differences in scale. *z* Scores with positive or negative values were considered positive or negative factors, respectively. A positive factor indicates that racial and ethnic group is doing better than White adults on that component. The primary factors were defined as the 1 or 2 components with the largest absolute *z* scores. A radar chart was then used to visualize and compare the relative magnitude by race and ethnicity. Subgroup analyses were stratified by sex and age (20-40, 41-60, and >60 years), and sensitivity analysis adjusting for age, sex, family income-to-poverty ratio, education level, marital status, insurance status, medication use for hypertension, diabetes, hyperlipidemia, and the presence of depression.

All analyses were performed using R software version 4.3.3 (R Project for Statistical Computing). The dietaryindex package was used to calculate the DASH diet score.^19^ A 2-sided *P* value less than .05 indicated a significant difference. The analysis was performed between March and October 2024.

## Results

### Characteristics of Study Population

The analysis included 16 104 nonpregnant adults aged 20 to 79 years from the NHANES 2011 to 2020 dataset (eFigure 1 in Supplement 1). The median (IQR) age of the study population was 46 (32-59) years, with 8262 participants (51.1%) being women. In terms of racial and ethnic composition, 1974 (5.2%) were Asian, 3918 (10.9%) were Black, 4144 (15.7%) were Latino/Hispanic, and 6068 (68.2%) were White adults. Compared with White individuals, Asian, Black, and Latino/Hispanic individuals were generally younger. Additionally, Black and Latino/Hispanic individuals had lower income and education levels and were less likely to have health insurance. Black individuals exhibited the highest rates of antihypertensive (1520 participants [33.83%]) and antidiabetic (467 participants [10.14%]) medication use, as well as the highest prevalence of depression (365 participants [9.72%]), whereas White individuals had the highest rate of lipid-lowering medication use (911 participants [15.32%]) (Table 1).

**Table 1.** Basic Characteristics by Race and Ethnicity in National Health and Nutrition Examination Survey 2011 to 2020

Characteristic	Participants, No. (%)^a^				*P* value
	Asian	Black	Latino/Hispanic	White	
Sample size	1974 (5.2)	3918 (10.9)	4144 (15.7)	6068 (68.2)	NA
Population size, No.	11 067 640	23 162 248	33 533 230	145 325 656	NA
Age, median (IQR)	41 (30-55)	43 (31-56)	39 (29-51)	49 (34-61)	<.001
Sex					
Male	983 (48.23)	1876 (45.64)	1993 (51.37)	2990 (48.91)	.001
Female	991 (51.77)	2042 (54.36)	2151 (48.63)	3078 (51.09)	
Ratio of family income to poverty					
≤1.30	310 (15.97)	1260 (34.68)	1479 (38.09)	1466 (13.88)	<.001
1.31-1.85	181 (9.37)	482 (13.45)	548 (14.24)	669 (8.14)	
1.86-3.50	418 (22.06)	905 (25.89)	934 (25.49)	1306 (23.83)	
>3.50	935 (52.61)	917 (25.98)	776 (22.18)	2356 (54.15)	
Education					
Less than high school	212 (9.68)	606 (14.07)	1621 (33.40)	646 (7.14)	<.001
High school	249 (12.59)	1077 (27.79)	899 (24.58)	1383 (22.09)	
Greater than high school	1512 (77.73)	2234 (58.14)	1623 (42.02)	4039 (70.77)	
Marital status					
Unmarried	575 (30.52)	2201 (56.52)	1435 (34.73)	2226 (32.87)	<.001
Married or living with partner	1398 (69.48)	1716 (43.48)	2706 (65.27)	3842 (67.13)	
Insurance					
Uninsured	282 (12.92)	803 (22.18)	1400 (36.72)	924 (10.96)	<.001
Insured	1689 (87.08)	3111 (77.82)	2733 (63.28)	5140 (89.04)	
Use of antihypertensive medication	366 (18.04)	1520 (33.83)	964 (16.96)	1602 (25.45)	<.001
Use of antidiabetic medication	158 (7.38)	467 (10.14)	438 (7.49)	447 (6.59)	<.001
Use of lipid-lowering medication	244 (11.62)	572 (12.23)	528 (8.46)	911 (15.32)	<.001
Depression	94 (5.33)	365 (9.72)	389 (8.80)	512 (6.93)	<.001

Abbreviation: NA, not applicable.

^a^ Percentages are weighted.

### Trends in Racial and Ethnic Differences in LE8 Scores

**Figure 1.**
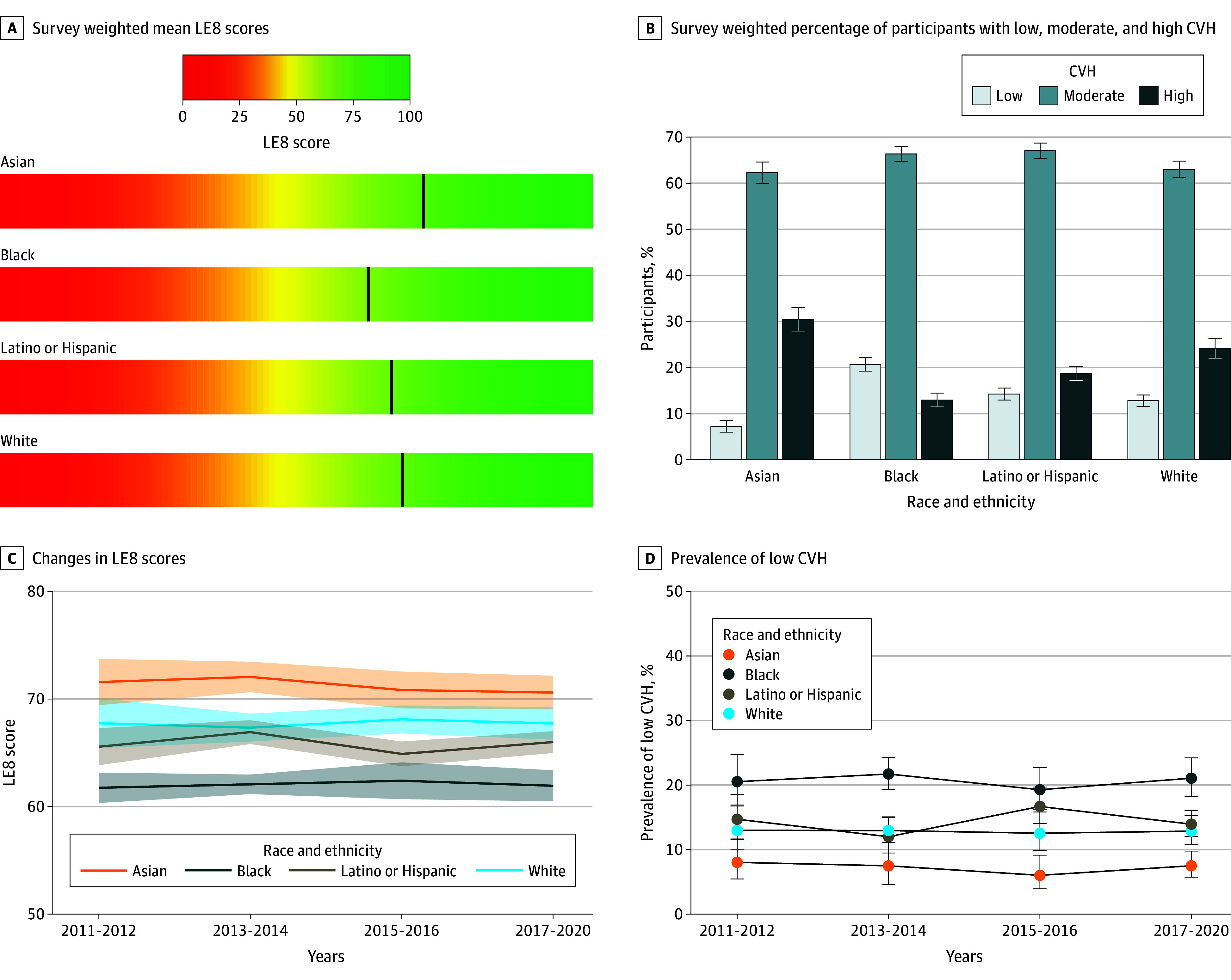
Distribution and Trends of Life’s Essential 8 (LE8) Score and Cardiovascular Health (CVH) Status A, Survey weighted mean LE8 scores across race and ethnicity groups. B, Survey weighted percentage of low cardiovascular health (CVH; LE8 score <50), moderate CVH (LE8 score 50-79), and high CVH (LE8 score ≥80) with error bars indicating 95% CI. C, Trends of LE8 scores by race and ethnicity. D, Weighted percentage with error bars indicating 95% CI of participants with low CVH (LE8 score <50) by race and ethnicity.

The overall LE8 scores stratified by race and ethnicity are presented in Figure 1A and eTable 2 in Supplement 1. Asian individuals had the highest mean LE8 score (71.2; 95% CI, 70.3-72.0), followed by White individuals (67.7; 95% CI, 66.9-68.6), Latino/Hispanic individuals (65.9; 95% CI, 65.2-66.5), and Black individuals (62.0; 95% CI, 61.3-62.7).

The distribution of CVH classes stratified by race and ethnicity are presented in Figure 1B and eTable 3 in Supplement 1. The prevalence of low CVH was lowest among Asian individuals at 7.3% (95% CI, 6.0%-8.5%), while Black individuals had the highest prevalence at 20.7% (95% CI, 19.2%-22.2%).

Between 2011 and 2020, the racial and ethnic differences in LE8 scores (Figure 1C; eTable 2 in Supplement 1) and prevalence of low CVH (Figure 1D; eTable 3 in Supplement 1) did not show significant change. As shown in Table 2, Black individuals consistently had lower scores than White individuals in both the 2011 to 2012 (mean, −6.01; 95% CI, −8.72 to −3.29; *P* < .001) and 2017 to 2020 cycles (mean, −5.79; 95% CI, −7.88 to −3.71; *P* < .001), with no significant change in the difference (dDoD, 0.21; 95% CI, −3.38 to 3.80; *P* = .91). In contrast, Asian individuals consistently had higher LE8 scores than White individuals in both the 2011 to 2012 (mean, 3.83; 95% CI, 0.67 to 6.99; *P* = .02) and 2017 to 2020 cycles (mean, 2.87; 95% CI, 0.70 to 5.05; *P* = .01), the gap was not significantly changed over time (dDoD, −0.95; 95% CI, −4.05 to 2.15; *P* = .54). No significant differences in LE8 scores were observed between Latino/Hispanic and White individuals in either the 2011 to 2012 or 2017 to 2020 survey cycles.

**Table 2.** Changes in Life’s Essential 8 (LE8) Score and Its Components by Race and Ethnicity^a^

LE8 component	Asian		Black		Latino/Hispanic	
	Mean (95% CI)	*P* value	Mean (95% CI)	*P* value	Mean (95% CI)	*P* value
LE8 score						
Difference with White, 2011 to 2012^b^	3.83 (0.67 to 6.99)	.02	−6.01 (−8.72 to −3.29)	<.001	−2.19 (−5.07 to 0.70)	.14
Difference with White, 2017 to 2020	2.87 (0.70 to 5.05)	.01	−5.79 (−7.88 to −3.71)	<.001	−1.73 (−3.54 to 0.08)	.06
dDoD^c^	−0.95 (−4.05 to 2.15)	.54	0.21 (−3.38 to 3.80)	.91	0.46 (−2.34 to 3.25)	.75
Diet						
Difference with White, 2011 to 2012	6.29 (1.61 to 10.97)	.01	−9.87 (−15.43 to −4.32)	<.001	−4.16 (−8.71 to 0.40)	.07
Difference with White, 2017 to 2020	12.57 (7.55 to 17.59)	<.001	−7.87 (−11.96 to −3.78)	<.001	−0.08 (−4.38 to 4.22)	.97
dDoD	6.27 (−0.53 to 13.08)	.07	2.00 (−5.17 to 9.16)	.58	4.08 (−2.35 to 10.51)	.21
Physical activity						
Difference with White, 2011 to 2012	−2.44 (−9.76 to 4.88)	.51	−9.37 (−16.42 to −2.33)	.01	−13.35 (−19.96 to −6.73)	<.001
Difference with White, 2017 to 2020	0.67 (−5.91 to 7.24)	.84	−8.48 (−13.13 to −3.82)	<.001	−8.70 (−13.08 to −4.32)	<.001
dDoD	3.11 (−5.38 to 11.59)	.47	0.90 (−7.39 to 9.19)	.83	4.64 (−2.25 to 11.53)	.18
Nicotine exposure						
Difference with White, 2011 to 2012	14.54 (11.04 to 18.03)	<.001	1.61 (−3.26 to 6.49)	.52	5.15 (1.52 to 8.78)	.01
Difference with White, 2017 to 2020	15.78 (11.42 to 20.15)	<.001	−0.42 (−5.37 to 4.52)	.87	5.03 (1.48 to 8.58)	.01
dDoD	1.24 (−4.77 to 7.25)	.68	−2.04 (−8.17 to 4.09)	.51	−0.12 (−5.54 to 5.30)	.96
Sleep health						
Difference with White, 2011 to 2012	1.29 (−1.41 to 4.00)	.35	−8.73 (−11.75 to −5.70)	<.001	−1.25 (−3.89 to 1.39)	.35
Difference with White, 2017 to 2020	−0.15 (−2.61 to 2.31)	.90	−10.94 (−13.2 to −8.68)	<.001	−4.38 (−6.53 to −2.22)	<.001
dDoD	−1.45 (−4.61 to 1.72)	.36	−2.21 (−5.44 to 1.02)	.18	−3.12 (−5.83 to −0.42)	.02
Body mass index^d^						
Difference with White, 2011 to 2012	1.37 (−3.49 to 6.22)	.58	−11.07 (−14.67 to −7.48)	<.001	−6.47 (−10.44 to −2.51)	.001
Difference with White, 2017 to 2020	−4.54 (−8.02 to −1.07)	.01	−6.74 (−10.1 to −3.37)	<.001	−4.63 (−7.83 to −1.42)	.01
dDoD	−5.91 (−11.65 to −0.18)	.04	4.34 (−1.15 to 9.83)	.12	1.85 (−2.59 to 6.28)	.41
Blood glucose						
Difference with White, 2011 to 2012	−3.57 (−7.32 to 0.19)	.06	−9.49 (−12.52 to −6.47)	<.001	−4.14 (−6.66 to −1.63)	.001
Difference with White, 2017 to 2020	−6.50 (−9.72 to −3.28)	<.001	−8.08 (−10.36 to −5.80)	<.001	−4.37 (−6.44 to −2.31)	<.001
dDoD	−2.93 (−7.84 to 1.98)	.24	1.41 (−2.71 to 5.54)	.49	−0.23 (−3.31 to 2.85)	.88
Blood lipids						
Difference with White, 2011 to 2012	5.35 (1.74 to 8.96)	.004	6.54 (3.60 to 9.48)	<.001	1.09 (−2.45 to 4.63)	.55
Difference with White, 2017 to 2020	−1.90 (−5.58 to 1.79)	.31	7.11 (4.04 to 10.18)	<.001	0.14 (−3.18 to 3.45)	.93
dDoD	−7.24 (−11.94 to −2.55)	.003	0.57 (−3.01 to 4.15)	.75	−0.95 (−6.05 to 4.14)	.71
Blood pressure						
Difference with White, 2011 to 2012	5.67 (1.99 to 9.35)	.003	−6.17 (−10.18 to −2.17)	.003	6.04 (2.01 to 10.08)	.003
Difference with White, 2017 to 2020	−0.49 (−3.39 to 2.41)	.74	−9.69 (−12.65 to −6.74)	<.001	2.26 (−0.68 to 5.20)	.13
dDoD	−6.16 (−10.21 to −2.11)	.004	−3.52 (−8.81 to 1.77)	.19	−3.78 (−8.53 to 0.97)	.12

Abbreviation: dDoD, descriptive difference-of-differences.

^a^ Non-Hispanic White adults served as the reference group.

^b^ The difference in LE8 score with White individuals was calculated by subtracting the LE8 values of White adults from those of Asian, Black, and Latino/Hispanic adults for both the 2011 to 2012 and 2017 to 2020 survey cycles.

^c^ Calculated using survey-weighted regression models that included an interaction term between survey year and race and ethnicity group.

^d^ Calculated as weight in kilograms divided by height in meters squared.

### Trends in Racial and Ethnic Differences in Individual Components of LE8

The individual LE8 component scores, stratified by race and ethnicity and survey year, are presented in eFigure 2 and eTable 4 in Supplement 1. Significant racial and ethnic differences were observed across several individual components of the LE8 score during the study period. Black individuals scored lower on most LE8 components compared with White individuals, except for blood lipids and nicotine exposure in 2011 to 2012. Notably, the Black vs White gap in LE8 components did not significantly change from 2011 to 2020 for any component (Table 2). The dDoD estimates were 2.00 (95% CI, −5.17 to 9.16; *P* = .58) for diet, 0.90 (95% CI, −7.39 to 9.19; *P* = .83) for physical activity, −2.04 (95% CI, −8.17 to 4.09; *P* = .51) for nicotine exposure, −2.21 (95% CI, −5.44 to 1.02; *P* = .18) for sleep health, 4.34 (95% CI, −1.15 to 9.83; *P* = .12) for BMI, 1.41 (95% CI, −2.71 to 5.54; *P* = .49) for blood glucose, 0.57 (95% CI, −3.01 to 4.15; *P* = .75) for blood lipids, and −3.52 (95% CI, −8.81 to 1.77; *P* = .19) for blood pressure.

Latino/Hispanic individuals had significantly lower physical activity, BMI, and blood glucose scores than White individuals in 2011 to 2012, but higher nicotine exposure and blood pressure scores. These differences remained stable over time, except for sleep health, where the gap widened significantly from 2011 to 2012 to 2017 to 2020 (dDoD, −3.12; 95% CI, −5.83 to −0.42; *P* = .02) (Table 2).

Asian individuals had higher scores in several LE8 components compared with White individuals in the 2011 to 2012 survey cycle, including diet, nicotine exposure, blood lipids, and blood pressure. Over time, the Asian vs White differences in blood lipids (Table 2) (dDoD, −7.24; 95% CI, −11.94 to −2.55; *P* = .003) and blood pressure (dDoD, −6.16; 95% CI, −10.21 to −2.11; *P* = .004) narrowed significantly and were no longer significant by the 2017 to 2020 cycle. Furthermore, BMI score differences reversed from being nonsignificant in 2011 to 2012 to significantly lower among Asian individuals compared with White individuals in 2017 to 2020 (Table 2) (dDoD, −5.91; 95% CI, −11.65 to −0.18; *P* = .04).

### Key Factors Associated With Racial and Ethnic Differences in LE8 Scores

**Figure 2.**
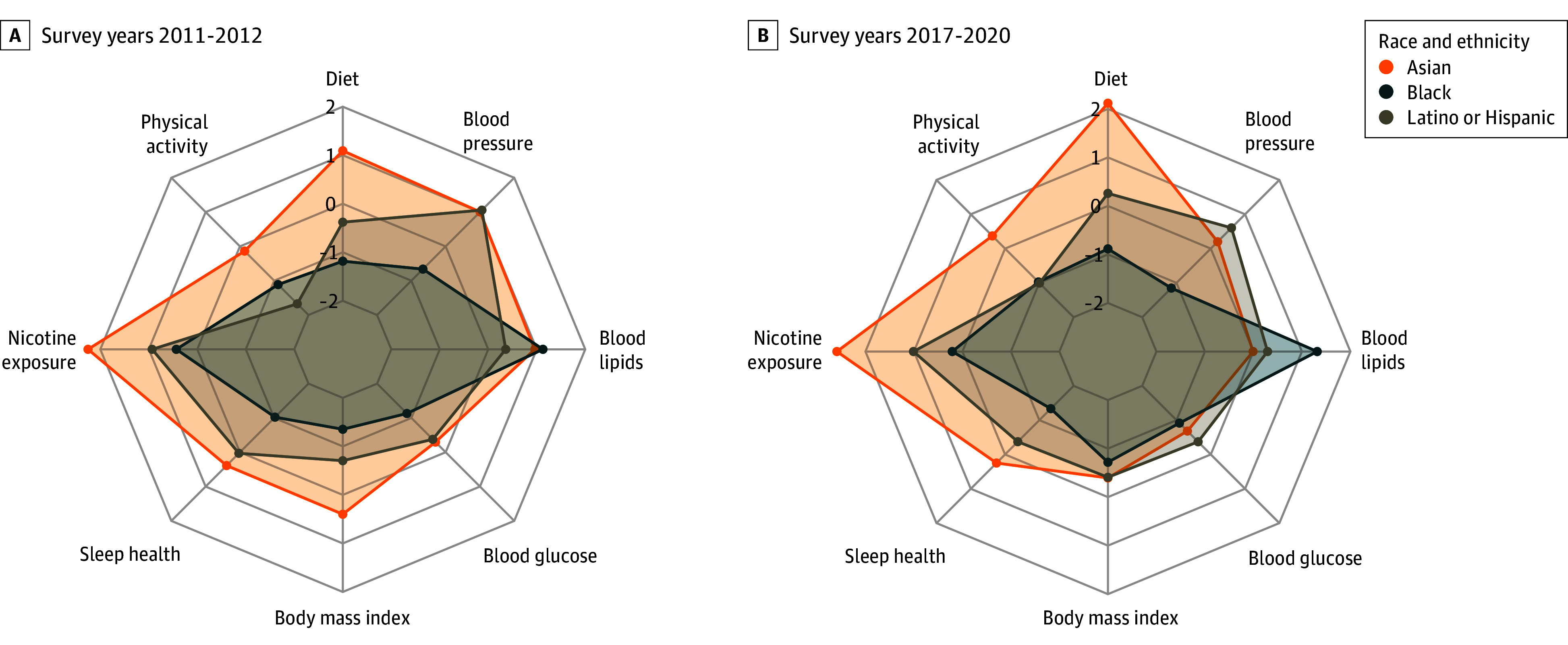
Relative Magnitude of Race and Ethnicity Difference in Each Life’s Essential 8 (LE8) Component The difference in each component of LE8 score compared with White individuals was calculated by subtracting the LE8 values of White adults from those of Asian, Black, and Latino/Hispanic adults for both the 2011 to 2012 (A) and 2017 to 2020 (B) survey cycles. *z* Scores were obtained by standardizing the values of all components across all racial and ethnic groups.

In the Black vs White disparity, most LE8 components were negative factors associated with the LE8 score gap in 2011 to 2012, with the exceptions of blood lipids and nicotine exposure. These relative associations remained largely unchanged in 2017 to 2020 (Figure 2; eTable 5 in Supplement 1). For Latino/Hispanic vs White differences, key factors in 2011 to 2012 included nicotine exposure and blood pressure (positive factors) and physical activity (a negative factor). By 2017 to 2020, the relative advantage in blood pressure had diminished, while other factors remained consistent. For Asian individuals, nicotine exposure was the greatest positive factor associated with higher LE8 scores in 2011 to 2012, while physical activity and blood glucose were negative factors. By 2017 to 2020, diet also became a major positive factor alongside nicotine exposure; however, the advantages in BMI, blood lipids, and blood pressure had diminished or even reversed.

### Subgroup Analyses of Trends in Racial and Ethnic Differences in Overall LE8 Score and Its Components

Subgroup analyses by sex and age revealed consistent trends in overall LE8 scores and in several individual components, such as diet, physical activity, nicotine exposure, and blood glucose levels, when comparing non-White groups to White individuals (eTables 6-14 in Supplement 1). For example, consistent with the main findings, the Black vs White difference in LE8 score did not change significantly from 2011 to 2012 to 2017 to 2020 in any subgroup. The dDoD estimates were 1.63 (95% CI, −2.02 to 5.28; *P* = .37) for male adults, −1.01 (95% CI, −5.40 to 3.38; *P* = .65) for female adults, 3.45 (95% CI, −0.69 to 7.59; *P* = .10) for those aged 20 to 40 years, −3.41 (95% CI, −7.92 to 1.11; *P* = .14) for those aged 41 to 60 years, and 1.56 (95% CI, −3.50 to 6.63; *P* = .54) for those older than 60 years. However, notable subgroup-specific differences emerged: the sleep health gap worsened significantly among young adults aged 20 to 40 across all Asian, Black, and Latino/Hispanic groups compared with White adults. The dDoD estimates were −4.61 (95% CI, −8.49 to −0.73; *P* = .02) for Asian adults; −4.70 (95% CI, −9.31 to v0.08; *P* = .04) for Black adults; −6.13 (95% CI, −9.74 to −2.52; *P* = .001) for Latino/Hispanic adults.

The Black vs White BMI score gap significantly narrowed among young adults (dDoD, 11.68; 95% CI, 3.20 to 20.15; *P* = .01), whereas the blood pressure gap widened among females (dDoD, −5.60; 95% CI, −11.17 to −0.03; *P* = .04) and middle-aged individuals (41-60 years; dDoD, −7.36; 95% CI, −12.91 to −1.80; *P* = .01). Among Asian subgroups, advantages previously observed in 2011 to 2012 diminished or reversed into disadvantages by 2017 to 2020. Specifically, BMI scores reversed among Asian males (dDoD, −8.46; 95% CI, −15.78 to −1.13; *P* = .02) and young adults (dDoD, −8.60; 95% CI, −16.91 to −0.28; *P* = .04), blood lipid scores decreased significantly among Asian females (dDoD, −5.69; 95% CI, −11.29 to −0.08; *P* = .04) and those aged 41 to 60 years (dDoD, −8.60; 95% CI, −15.61 to −1.59; *P* = .02), and blood pressure scores notably worsened among Asian females (dDoD, −6.99; 95% CI, −11.51 to −2.47; *P* = .003) and adults over 60 years (dDoD, −9.44; 95% CI, −18.74 to −0.13; *P* = .04).

The relative magnitude of race and ethnicity difference in each LE8 components across sex and age groups are further detailed in eFigures 3 and 4 in Supplement 1. Notably, nicotine exposure was a positive factor to Black vs White differences predominantly among males and younger adults (aged ≤40 years). For Latino/Hispanic vs White differences, blood lipids, alongside nicotine exposure, emerged as key positive factors among older adults (>60 years) in 2017 to 2020. Among Asians compared with the White group, the advantage in lower nicotine exposure was more pronounced among female participants but diminished in older adults.

### Sensitivity Analysis of Trends in Racial and Ethnic Differences in Overall LE8 Score and Its Components

After adjustment for these confounding factors, including psychological health, the primary findings remained largely consistent. Specifically, the Black vs White differences in LE8 scores were unchanged (eTable 15 in Supplement 1). However, while the Hispanic vs White sleep health gap (dDoD, −2.44; 95% CI, −5.32 to 0.44; *P* = .09) and the Asian vs White BMI gap (dDoD, −4.45; 95% CI, −11.47 to 2.57; *P* = .21) showed trends similar to the main results, these differences were nonsignificant. Importantly, the relative magnitude of race and ethnicity difference in each LE8 component across racial and ethnic differences remained robust after adjusting for these psychological and sociodemographic factors (eFigure 5 in Supplement 1).

## Discussion

This study examined trends in racial and ethnic differences in LE8 scores among US adults from 2011 to 2020, along with the key components associated with these disparities. Our results revealed that Asian adults consistently had the highest LE8 scores and lowest rates of low CVH, while Black adults had the lowest LE8 scores and highest rates of low CVH. These differences remained stable over the decade, with no significant overall improvement. However, we found evolving trends in individual LE8 components, including declines in BMI, blood lipids, and blood pressure scores among Asian individuals, and a widening gap in sleep health between Latino/Hispanic and White individuals. Black vs White differences in LE8 scores remained stable, with the gap largely associated with components other than blood lipids and nicotine exposure.

Our study extends the prior literature in 3 important ways. First, while previous research has consistently shown significant racial and ethnic disparities in CVH,^20,21^ our study focuses on the updated LE8 framework, offering a more comprehensive analysis of CVH that includes sleep health.^7^ Importantly, we analyzed trends in racial differences in CVH using a statistical approach, demonstrating that these differences persisted throughout the decade, particularly among Black adults, supplementing findings from prior studies.^8,9^

Second, unlike most previous studies that focused on overall CVH or limited components of health,^5,20,21^ we provide a detailed breakdown of racial and ethnic differences across all LE8 components. Our findings expand on previous studies by thoroughly analyzing the racial and ethnic differences for each LE8 component. Notably, we identified a worsening sleep health gap in the Latino/Hispanic population compared with White individuals. Moreover, subgroup analyses indicate that this decline in sleep health was evident only among younger adults. Additionally, the observed reversal in BMI advantages and the diminishing differences in blood lipids and blood pressure among Asian individuals, particularly within specific subgroups, provide a more detailed understanding of how health disparities manifest across various aspects of CVH.

Third, this study uniquely assesses the relative magnitude of race and ethnicity difference in each LE8 component, thereby identifying specific areas where targeted interventions could be most effective. While socioeconomic factors have been cited as drivers of these disparities,^22,23^ our analysis goes further by quantifying the relative magnitude of race and ethnicity difference in each component, with particular emphasis on the roles of physical activity in Latino/Hispanic vs White differences, and diet and nicotine exposure in Asian vs White differences. This provides a more granular understanding compared with prior studies, which often treated these components collectively.

Our findings have significant implications for public health and clinical practice. Despite extensive public health efforts to address health disparities, including health care reforms like the Affordable Care Act,^24,25^ racial and ethnic gaps in CVH have not markedly improved over the past decade. This highlights the need for more effective, tailored interventions that target specific drivers of these differences. For example, Black vs White differences associated with multiple factors, including diet, physical activity, and sleep health, suggesting that culturally tailored outreach, enhanced access to preventive services, and behavioral support could be more effective than a one-size-fits-all approach. Similarly, the decline in health indicators like BMI, blood lipids, and blood pressure among Asian individuals calls for early, culturally appropriate interventions to prevent further deterioration. The widening sleep health gap among Latino/Hispanic individuals and younger adults also underscores the need to prioritize underrecognized contributors to CVH, such as sleep, in public health initiatives. Addressing sleep health differences may involve tackling broader social determinants like work conditions and stress,^26^ which disproportionately affect Latino/Hispanic communities. Public health strategies need to be adapted to meet the unique needs of each racial and ethnic group.

### Limitations

Our study has several limitations. First, behavioral variables were self-reported, which may introduce bias. However, these measures have been widely used in previous research. Second, fasting plasma glucose measurements were only available for a subset of participants, limiting the generalizability of findings related to blood glucose. Third, our findings are only applicable to non-Hispanic Asian, Black, Latino/Hispanic, and White general populations, as we did not include or analyze other racial and ethnic groups separately. Future studies should include underrepresented groups to validate broader applicability. Additionally, while NHANES employs strategies to reduce bias from nonresponse, residual effects may persist. To mitigate this, we adjusted for survey design and applied sample weights to account for nonresponse bias.

## Conclusions

In conclusion, this study underscores the persistent racial and ethnic differences in LE8 scores over the past decade. Targeted, effective interventions are necessary to address the key factors associated with these differences and improve CVH equity.

## Notes

Supplement 1. **eMethods. **Detailed Description of LE8 Calculation and Covariates **eFigure 1. **Flowchart Illustrating Participant Selection **eFigure 2. **Trends of Each Component in Life's Essential 8 Score by Race and Ethnicity **eFigure 3. **Subgroup Analyses of Relative Magnitude of Race and Ethnicity Difference in Each Life's Essential 8 Component According to Sex **eFigure 4. **Subgroup Analyses of Relative Magnitude of Race and Ethnicity Difference in Each Life's Essential 8 Component According to Age **eFigure 5. **Relative Magnitude of Race and Ethnicity Difference in Each Life's Essential 8 Component after Adjusted for Confounding Factors **eTable 1. **Definition of Sociodemographic and Scoring Approach for Quantifying Life’s Essential 8 in the National Health and Nutrition Examination Surveys, 2011-2020 **eTable 2. **Overall Life's Essential 8 Scores and Scores Stratified by Race/Ethnicity and Survey Year **eTable 3. **Prevalence of low cardiovascular health (LE8 score <50) **eTable 4. **Each Component of Life’s Essential 8 Score by Race and Ethnicity and Survey Year **eTable 5. **Z-Scores for Racial Differences in Life's Essential 8 Components **eTable 6. **Subgroup Analysis of Changes in Life's Essential 8 Score by Race and Ethnicity According to Sex and Age **eTable 7. **Subgroup Analysis of Changes in Diet Score by Race and Ethnicity According to Sex and Age **eTable 8. **Subgroup Analysis of Changes in Activity Score by Race and Ethnicity According to Sex and Age **eTable 9. **Subgroup Analysis of Changes in Nicotine Exposure Score by Race and Ethnicity According to Sex and Age **eTable 10. **Subgroup Analysis of Changes in Sleep Health Score by Race and Ethnicity According to Sex and Age **eTable 11. **Subgroup Analysis of Changes in Body Mass Index Score by Race and Ethnicity According to Sex and Age **eTable 12. **Subgroup Analysis of Changes in Blood Glucose Score by Race and Ethnicity According to Sex and Age **eTable 13. **Subgroup Analysis of Changes in Blood Lipids Score by Race and Ethnicity According to Sex and Age **eTable 14. **Subgroup Analysis of Changes in Blood Pressure Score by Race and Ethnicity According to Sex and Age

Supplement 2.
Data Sharing Statement
