# Supplementary material for: Trends and Key Factors Associated With Racial and Ethnic Differences in Life’s Essential 8 Scores
Source: JAMA Netw Open. 2025 Jun 18;8(6):e2516663. doi: 10.1001/jamanetworkopen.2025.16663 (PMC12177675; doi:10.1001/jamanetworkopen.2025.16663)
Supplement: Supplement 1. — eMethods. Detailed Description of LE8 Calculation and Covariates eFigure 1. Flowchart Illustrating Participant Selection eFigure 2. Trends of Each Component in Life's Essential 8 Score by Race and Ethnicity eFigure 3. Subgroup Analyses of Relative Magnitude of Race and Ethnicity Difference in Each Life's Essential 8 Component According to Sex eFigure 4. Subgroup Analyses of Relative Magnitude of Race and Ethnicity Difference in Each Life's Essential 8 Component According to Age eFigure 5. Relative Magnitude of Race and Ethnicity Difference in Each Life's Essential 8 Component after Adjusted for Confounding Factors eTable 1. Definition of Sociodemographic and Scoring Approach for Quantifying Life’s Essential 8 in the National Health and Nutrition Examination Surveys, 2011-2020 eTable 2. Overall Life's Essential 8 Scores and Scores Stratified by Race/Ethnicity and Survey Year eTable 3. Prevalence of low cardiovascular health (LE8 score <50) eTable 4. Each Component of Life’s Essential 8 Score by Race and Ethnicity and Survey Year eTable 5. Z-Scores for Racial Differences in Life's Essential 8 Components eTable 6. Subgroup Analysis of Changes in Life's Essential 8 Score by Race and Ethnicity According to Sex and Age eTable 7. Subgroup Analysis of Changes in Diet Score by Race and Ethnicity According to Sex and Age eTable 8. Subgroup Analysis of Changes in Activity Score by Race and Ethnicity According to Sex and Age eTable 9. Subgroup Analysis of Changes in Nicotine Exposure Score by Race and Ethnicity According to Sex and Age eTable 10. Subgroup Analysis of Changes in Sleep Health Score by Race and Ethnicity According to Sex and Age eTable 11. Subgroup Analysis of Changes in Body Mass Index Score by Race and Ethnicity According to Sex and Age eTable 12. Subgroup Analysis of Changes in Blood Glucose Score by Race and Ethnicity According to Sex and Age eTable 13. Subgroup Analysis of Changes in Blood Lipids Score by Race and Ethnicity According to Sex and Age eTable 14. [file jamanetwopen-e2516663-s001.pdf]

Supplemental Online Content

Yang H, Huang C, Sawano M, et al. Trends and key contributors of racial and ethnic differences in Life's Essential 8 scores. *JAMA Netw Open*. 2025;8(6):e2516663.
doi:10.1001/jamanetworkopen.2025.16663

eMethods. Detailed Description of LE8 Calculation and Covariates
eFigure 1. Flowchart Illustrating Participant Selection
eFigure 2. Trends of Each Component in Life's Essential 8 Score by Race and Ethnicity
eFigure 3. Subgroup Analyses of Relative Magnitude of Race and Ethnicity Difference in Each Life's Essential 8 Component According to Sex
eFigure 4. Subgroup Analyses of Relative Magnitude of Race and Ethnicity Difference in Each Life's Essential 8 Component According to Age
eFigure 5. Relative Magnitude of Race and Ethnicity Difference in Each Life's Essential 8 Component after Adjusted for Confounding Factors
eTable 1. Definition of Sociodemographic and Scoring Approach for Quantifying Life's Essential 8 in the National Health and Nutrition Examination Surveys, 2011-2020
eTable 2. Overall Life's Essential 8 Scores and Scores Stratified by Race/Ethnicity and Survey Year
eTable 3. Prevalence of low cardiovascular health (LE8 score <50)
eTable 4. Each Component of Life's Essential 8 Score by Race and Ethnicity and Survey Year
eTable 5. Z-Scores for Racial Differences in Life's Essential 8 Components
eTable 6. Subgroup Analysis of Changes in Life's Essential 8 Score by Race and Ethnicity According to Sex and Age
eTable 7. Subgroup Analysis of Changes in Diet Score by Race and Ethnicity According to Sex and Age
eTable 8. Subgroup Analysis of Changes in Activity Score by Race and Ethnicity According to Sex and Age
eTable 9. Subgroup Analysis of Changes in Nicotine Exposure Score by Race and Ethnicity According to Sex and Age
eTable 10. Subgroup Analysis of Changes in Sleep Health Score by Race and Ethnicity According to Sex and Age
eTable 11. Subgroup Analysis of Changes in Body Mass Index Score by Race and Ethnicity According to Sex and Age
eTable 12. Subgroup Analysis of Changes in Blood Glucose Score by Race and Ethnicity According to Sex and Age
eTable 13. Subgroup Analysis of Changes in Blood Lipids Score by Race and Ethnicity According to Sex and Age
eTable 14. Subgroup Analysis of Changes in Blood Pressure Score by Race and Ethnicity According to Sex and Age

This supplemental material has been provided by the authors to give readers additional information about their work.

© 2025 Yang H et al. *JAMA Network Open*.

© 2025 Yang H et al. *JAMA Network Open*.

eMethods. Detailed Description of LE8 Calculation and Covariates.

LE8 Calculation

The assessment of LE8 score begins with a Dietary Approaches to Stop Hypertension (DASH)-style diet evaluated through the first 24-hour dietary recall, capturing intake from eight food and nutrient groups: fruits, whole grains, vegetables, nuts and legumes, low-fat dairy products, red and processed meats, sugar-sweetened beverages, and sodium. Each component is assigned between 1 to 5 points, accumulating up to a total of 40 points.

Health behaviors, gathered from interviews, include self-reported weekly minutes of moderate to vigorous physical activity, nicotine exposure (including use of cigarettes and secondhand smoke exposure), and average hours of sleep per night. Anthropometric measurements considered are body mass index (BMI), calculated as body weight divided by height squared, and blood pressure, which is averaged across second to fourth measurements depending on availability.

Laboratory indicators assessed include non-high density lipoprotein cholesterol (non-HDL-C, equal to total cholesterol minus HDL-C), fasting blood glucose and casual Hemoglobin A1c (HbA1c) levels. The overall LE8 score is the mean of these eight metrics, also ranging from 0 to 100. Based on AHA recommendations, LE8 scores are categorized into three levels of cardiovascular health: low CVH (score < 50), medium CVH (score 50-79), and high CVH (score ≥ 80).

Covariates

Demographic and socioeconomic information was collected through interviews, including race, age, sex, the ratio of family income to poverty, education level, marital status, and insurance status. The ratio of family income to poverty was defined based on the Department of Health and Human Services guidelines,[16] calculated as the ratio of monthly family income to poverty levels and classified into four categories: low income (≤1.30), lower middle income (1.31-1.85), middle income (1.86-3.50), and high income (>3.50). Education levels were segmented into less than high school, high school graduate, and

© 2025 Yang H et al. *JAMA Network Open*.

more than high school. Marital status was categorized as unmarried, and married or living with a partner. Insurance status was recorded as uninsured and insured.

Additionally, medication use for hypertension, diabetes (including oral medications and insulin), and hyperlipidemia was self-reported. Depression was assessed using the two-item Patient Health Questionnaire (PHQ-2), with a validated threshold score of ≥3 indicating depression.

© 2025 Yang H et al. *JAMA Network Open*.

eFigure 1. Flowchart Illustrating Participant Selection.

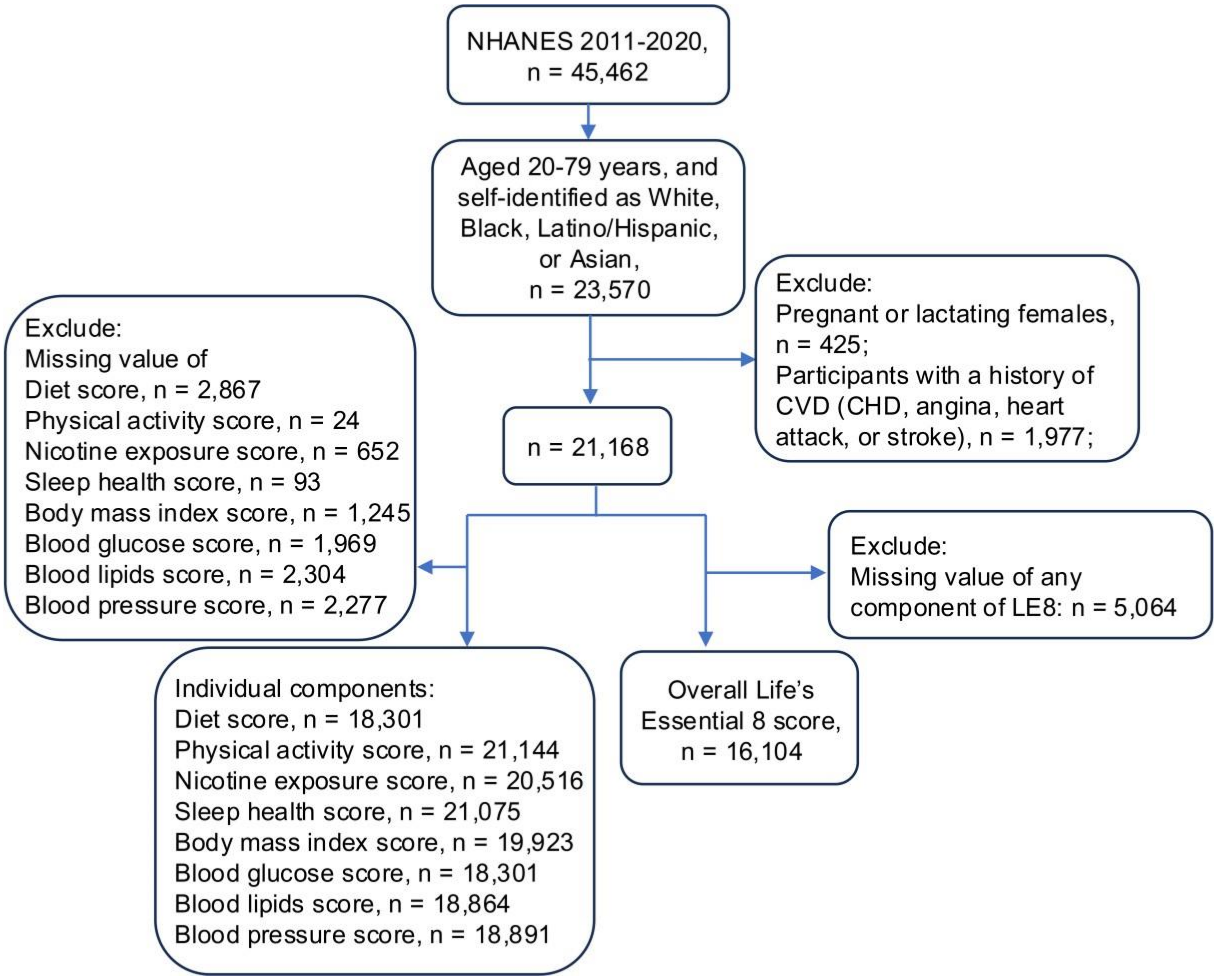

© 2025 Yang H et al. *JAMA Network Open*.

eFigure 2. Trends of Each Component in Life's Essential 8 Score by Race and Ethnicity.

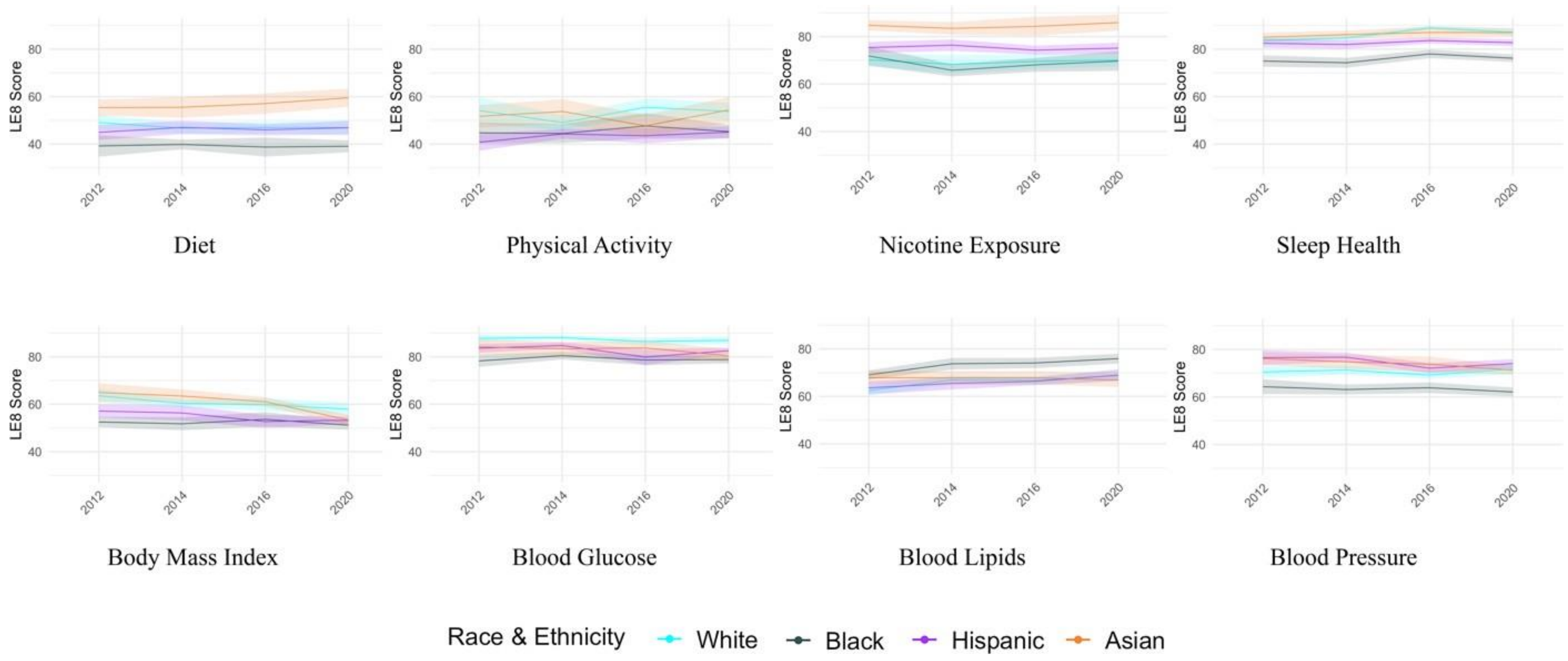

© 2025 Yang H et al. *JAMA Network Open*.

eFigure 3. Subgroup Analyses of Relative Contributions of Each Component in Life's Essential 8 to Racial and Ethnic Differences According to Sex.

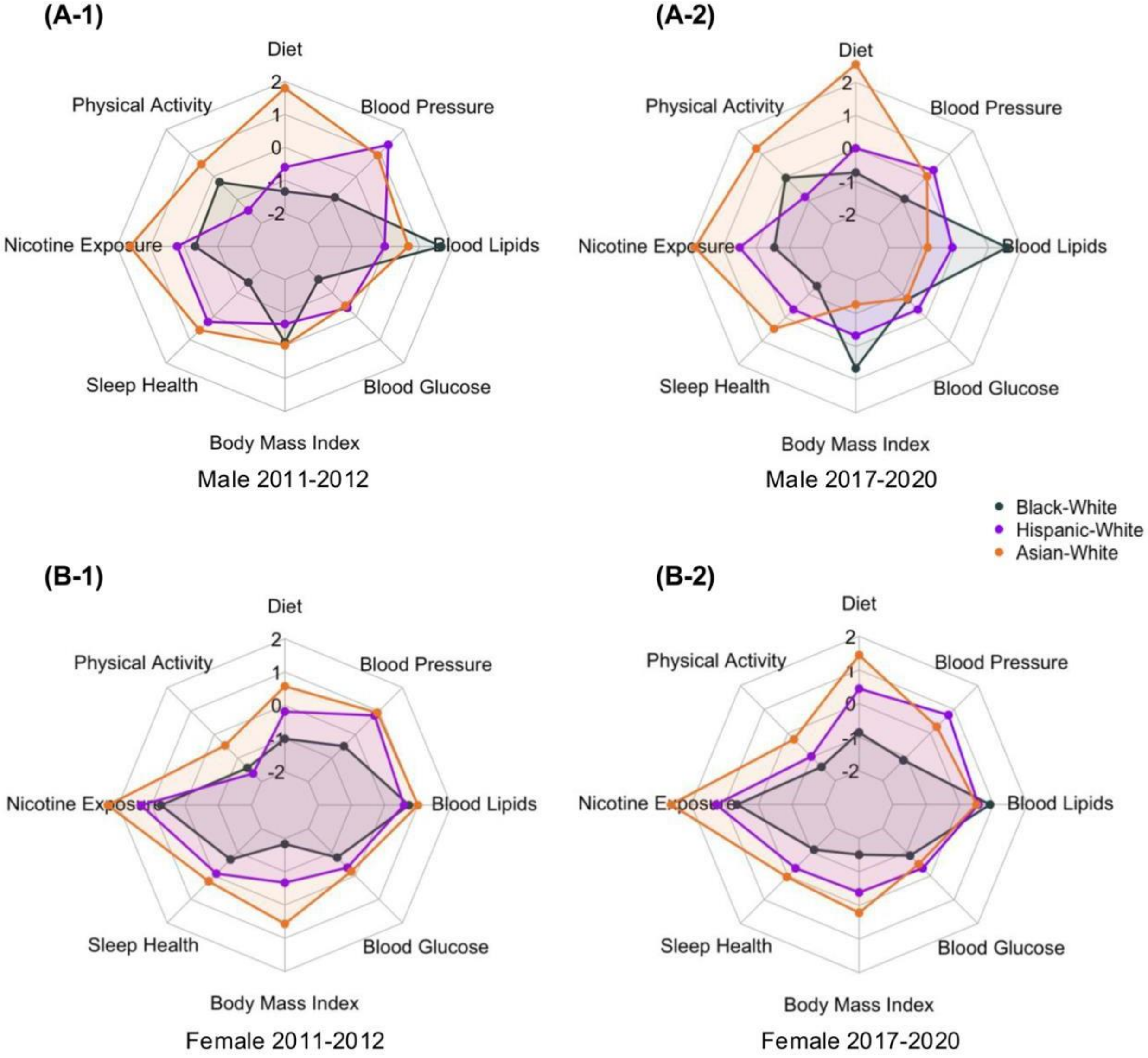

The difference in each component of Life's Essential 8 (LE8) score compared to White individuals was calculated by subtracting the LE8 values of White adults from those of Black, Latino/Hispanic, and Asian adults for both the 2011-2012 and 2017-2020 survey cycles. Z-scores were obtained by standardizing the values of all components across all racial and ethnic groups.

© 2025 Yang H et al. *JAMA Network Open*.

eFigure 4. Subgroup Analyses of Relative Contributions of Each Component in Life's Essential 8 to Racial and Ethnic Differences According to Age.

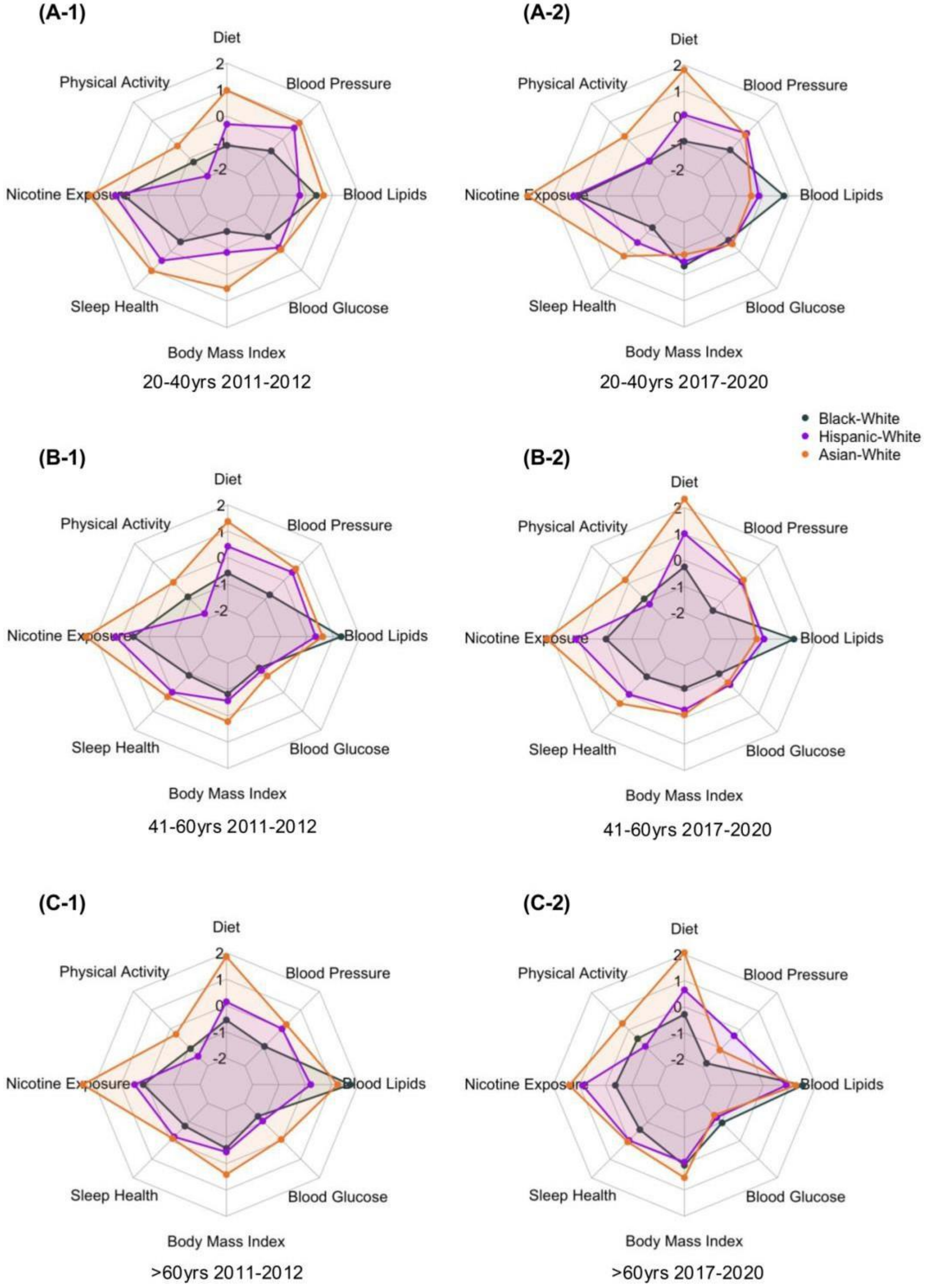

The difference in each component of Life's Essential 8 (LE8) score compared to White individuals was calculated by subtracting the LE8 values of White adults from those of Black, Latino/Hispanic, and Asian adults for both the 2011-2012 and 2017-2020 survey cycles. Z-scores were obtained by standardizing the values of all components across all racial and ethnic groups.

© 2025 Yang H et al. *JAMA Network Open*.

eFigure 5. Relative Contributions of Each Component in Life's Essential 8 to Racial and Ethnic Differences after Adjusted for Confounding Factors.

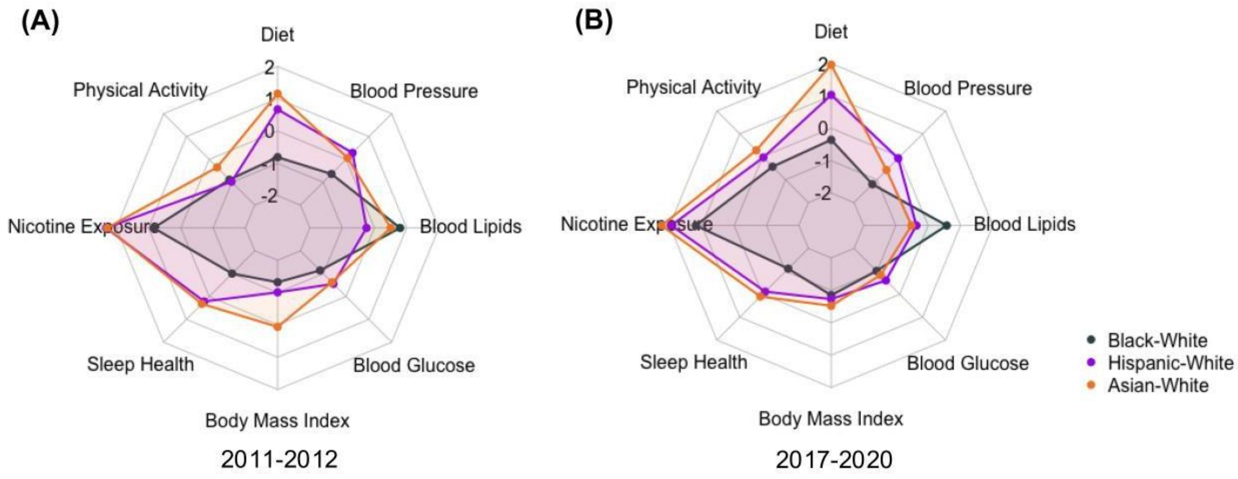

The difference in each component of Life's Essential 8 (LE8) score compared to White individuals was calculated by subtracting the LE8 values of White adults from those of Black, Latino/Hispanic, and Asian adults for both the 2011-2012 (A) and 2017-2020 (B) survey cycles. Z-scores were obtained by standardizing the values of all components across all racial and ethnic groups. Adjusted for age, sex (male, female), family income-to-poverty ratio (≤1.30, 1.31–1.85, 1.86–3.50, and >3.50), education level (less than high school, high school graduate, and more than high school), marital status (unmarried, married or living with a partner), insurance status (uninsured, insured), medication use for hypertension, diabetes, and hyperlipidemia, and the presence of depression.

© 2025 Yang H et al. *JAMA Network Open*.

eTable 1. Definition of Sociodemographic and Scoring Approach for Quantifying Life's Essential 8 in the National Health and Nutrition Examination Surveys, 2011-2020.

Risk factor	Ascertainment in NHANES	Definition of variables used in analysis
Ratio of family income to poverty	In-person interview: Please describe your family income (reported as a range value in dollars)	Ratio of family income to poverty was defined by the Department of Health and Human Services guidelines, was calculated as the ratio of monthly family income to poverty levels and classified into four categories: low income (≤1.30), lower middle income (1.31-1.85), middle income (1.86-3.50), and high income (>3.50).
Education level	In-person interview: What is the highest grade or level of school you have completed or the highest degree you have received?	Highest education level is classified as less than high school, high school, greater than high school.
Marital status	In-person interview: Please describe your current marital status.	Marital status is classified as married/ living with partners vs. unmarried (widowed, divorced, or separated; and never married).
Health insurance	In-person interview: Are you covered by health insurance or some other kind of health care plan? [Include health insurance obtained through employment or purchased directly as well as government programs like Medicare and Medicaid that provide medical care or help pay medical bills.]	Individuals are classified as insured if they had any private health insurance, Medicare, Medicaid, military plan, government or state-sponsored health plan.
DASH Diet	24-h recall dietary interview (day 1 and day 2): The first dietary recall interview is collected in-person in the Mobile Examination Center (MEC) and the second interview is collected by telephone 3 to 10 days later.	DASH diet score was calculated using the method of Frank B. H. et al.;<br><br>**Scoring (Population):**<br>Points Quantile<br>100 ≥95th %ile (top/ideal diet)<br>80 75th – 94th %ile<br>50 50th – 74th %ile<br>25 25th – 49th %ile<br>0 1st – 24th %ile (bottom/least ideal quartile)
Physical activity	In-person interview:<br>• Behavioral Risk Factor Surveillance Survey (BRFSS) physical activity instrument.<br>• Patients were asked if they participated in moderate or vigorous physical activity during the past 30 days. If they answered yes to either question, they were then asked the duration and frequency of their participation in physical activity for an average week.	Physical activity is calculated into recreational activities minutes per week: (Days moderate recreational activities in a typical week * Minutes moderate recreational activities on a typical day) + (Days vigorous recreational activities in a typical week * Minutes vigorous recreational activities on a typical day).

© 2025 Yang H et al. *JAMA Network Open*.

		**Metric:** Minutes of moderate (or greater) intensity activity per week<br>**Scoring:**<br>Points Minutes<br>100 ≥150<br>90 120 – 149<br>80 90 – 119<br>60 60 – 89<br>40 30 – 59<br>20 1 – 29<br>0 0
Nicotine exposure	In-person interview:<br>• Ever smoke cigarettes in entire life<br>• Do you now smoke cigarettes?<br>• How long has it been since you quit smoking cigarettes?<br>• How many people who live here smoke cigarettes, cigars, little cigars, pipes, water pipes, hookah, or any other tobacco product?	Never smokers are defined as individuals who stated never smokes.<br>Former smokers are defined as individuals who stated that they had smoked but now did not smoke.<br>Current smokers are defined as individuals who stated that they smoked cigarettes currently.<br>Living with active indoor smoker in home are defined as live with at least one people who smoke any tobacco product.<br><br>**Metric:** Combustible tobacco use and/or inhaled nicotine delivery systems (NDS) use; or secondhand smoke exposure<br>**Scoring:**<br>Points Status<br>100 Never smoker<br>75 Former smoker, quit ≥ 5 yrs<br>50 Former smoker, quit 1 to < 5 yrs<br>25 Former smoker, quit <1 year, or currently<br>using inhaled NDS<br>0 Current smoker<br>Subtract 20 points (unless score is 0) for living with active indoor smoker in home.
Sleep health	In-person interview:<br>Number of hours usually sleep on weekdays or workdays.	**Metric:** Average hours of sleep per night<br>**Scoring:**<br>Points Level<br>100 7 to < 9 h<br>90 9 to < 10 h<br>70 6 to < 7 h<br>40 5 to < 6 or ≥ 10 h<br>20 4 to <5 h<br>0 <4 h
Body mass index (kg/m^2)	Examination measurement:<br>Weight is measured on all examinees<br>Standing height is measured on all examinees 2 years and older	Body mass index (BMI, kg/m^2) are calculated as body weight (kg) divided by height squared (m^2).<br><br>**Metric:** BMI (kg/m^2)

© 2025 Yang H et al. *JAMA Network Open*.

		**Scoring:**<br>Points Level<br>100 <25<br>70 25.0 to 29.9<br>30 30.0 to 34.9<br>15 35.0 to 39.9<br>0 ≥ 40
Blood glucose & Diabetes	In-person interview:<br>• Are you now taking diabetic pills to lower your blood sugar? These are sometimes called oral agents or oral hypoglycemic agents.<br>• Are you now taking insulin?<br>• Self-reported use of prescription medications during a one-month period prior to the survey date.<br><br>Lab measurement:<br>All participants ages 12 and older are given the option of a HbA1C% test during their physical examination. Besides, Participants aged 12 years and older who were examined in the morning session were tested for fasting glucose.	Diabetes is defined based on fasting glucose ≥ 126 mg/dL (7.0 mmol/L), or HbA1c ≥ 6.5%, or currently on antidiabetic medication.<br><br>**Metric:** Fasting blood glucose (FBG, mg/dL) or Hemoglobin A1c (%)<br>**Scoring:**<br>Points Level<br>100 No history of diabetes and FBG <100 (or<br>HbA1c < 5.7%)<br>60 No diabetes and FBG 100 – 125 mg/dL (5.55 – 6.94 mmol/L) (or HbA1c 5.7-6.4%) (Pre-diabetes)<br>40 Diabetes with HbA1c <7.0%<br>30 Diabetes with HbA1c 7.0 – 7.9%<br>20 Diabetes with HbA1c 8.0 – 8.9%<br>10 Diabetes with Hb A1c 9.0 – 9.9%<br>0 Diabetes with HbA1c ≥10.0%
Blood lipids	Lab measurement:<br>All participants aged 6 years and older.	Non-HDL-cholesterol (mg/dL) was calculated by plasma total minus HDL-cholesterol.<br><br>**Metric:** Non-HDL-cholesterol (mg/dL)<br>**Scoring**:<br>Points Level<br>100 <130 mg/dL (3.36 mmol/L)<br>60 130 – 159 mg/dL (3.36 - 4.11 mmol/L)<br>40 160 – 189 mg/dL (4.14 - 4.89 mmol/L)<br>20 190 – 219 mg/dL (4.91 – 5.66 mmol/L)<br>0 ≥220 mg/dL (5.69 mmol/L)<br>If drug-treated level, subtract 20 points

© 2025 Yang H et al. *JAMA Network Open*.

Blood pressure & Hypertension	In-person interview:<br>Are you now taking prescribed medicine for high blood pressure (BP)?<br><br>Examination measurement:<br>Blood pressure (BP) is measured on all examinees 8 years and older.	Hypertension is defined as average systolic blood pressure ≥ 140, or averaged diastolic blood pressure ≥ 90, or currently on antihypertensive medication.<br><br>**Metric:** Systolic and diastolic blood pressure (mm Hg)<br>**Scoring:**<br>Points Level<br>100 <120/<80 (Optimal)<br>75 120-129/<80 (Elevated)<br>50 130-139 or 80-89 (Stage I HTN)<br>25 140-159 or 90-99<br>0 ≥160 or ≥100<br>Subtract 20 points (unless score is 0) if treated level

© 2025 Yang H et al. *JAMA Network Open*.

eTable 2. Overall Life's Essential 8 Scores and Scores Stratified by Race/Ethnicity and Survey Year.

	Total	Asian	Black	Hispanic	White	*P* value
**Le8 score**						
	16,104	1,974	3,918	4,144	6,068	
Total	67.0 (66.4, 67.6)	71.2 (70.3, 72.0)	62.0 (61.3, 62.7)	65.9 (65.2, 66.5)	67.7 (66.9, 68.6)	<0.001
	3,576	494	960	762	1,360	
2011-2012	67.0 (65.3, 68.6)	71.6 (69.4, 73.7)	61.7 (60.3, 63.2)	65.6 (63.8, 67.3)	67.7 (65.4, 70.1)	<0.001
	3,858	443	799	946	1,670	
2013-2014	66.9 (65.9, 68.0)	72.0 (70.6, 73.5)	62.1 (61.2, 63.0)	66.9 (65.8, 68.0)	67.3 (66.0, 68.6)	<0.001
	3,662	430	785	1,212	1,235	
2015-2016	67.1 (65.9, 68.4)	70.8 (69.1, 72.5)	62.4 (60.7, 64.1)	64.9 (63.7, 66.0)	68.1 (66.8, 69.4)	<0.001
	5,008	607	1,374	1,224	1,803	
2017-2020	67.0 (65.8, 68.1)	70.6 (69.0, 72.2)	61.9 (60.5, 63.4)	66.0 (65.0, 67.0)	67.7 (66.2, 69.2)	<0.001

Data are presented as sample size, and survey weighted mean with 95% confidence intervals (CIs).

© 2025 Yang H et al. *JAMA Network Open*.

eTable 3. Prevalence of low cardiovascular health (LE8 score <50).

	**Total**	**Asian**	**Black**	**Hispanic**	**White**	***P* value**
Total	13.6 (12.7, 14.6)	7.3 (6.0, 8.5)	20.7 (19.2, 22.2)	14.3 (13.0, 15.6)	12.8 (11.6, 14.1)	<0.001
2011-2012	13.8 (11.6, 16.1)	8.0 (5.5, 11.6)	20.5 (16.9, 24.7)	14.7 (11.5, 18.5)	13.0 (10.0, 16.7)	0.007
2013-2014	13.5 (12.3, 14.7)	7.5 (4.6, 12.1)	21.7 (19.4, 24.3)	12.0 (9.5, 15.1)	12.9 (11.1, 15.0)	<0.001
2015-2016	13.6 (11.2, 15.9)	6.0 (3.9, 9.1)	19.3 (16.2, 22.7)	16.7 (14.1, 19.7)	12.5 (9.8, 15.8)	<0.001
2017-2020	13.6 (12.0, 15.2)	7.5 (5.7, 9.8)	21.1 (18.2, 24.2)	13.9 (12.1, 16.1)	12.9 (10.8, 15.3)	<0.001

Data are presented as survey weighted prevalence with 95% confidence intervals (CIs).

© 2025 Yang H et al. *JAMA Network Open*.

eTable 4. Each Component of Life's Essential 8 Score by Race and Ethnicity and Survey Year.

	Total	Asian	Black	Hispanic	White	*P* value
**Diet**						
Total	46.8 (45.6, 47.9)	57.4 (55.2, 59.5)	39.2 (37.6, 40.9)	46.4 (44.9, 47.8)	47.4 (45.9, 48.9)	<0.001
2011-2012	47.7 (45.5, 49.9)	55.4 (52.0, 58.8)	39.2 (34.7, 43.7)	44.9 (41.7, 48.1)	49.1 (45.9, 52.3)	<0.001
2013-2014	46.5 (44.9, 48.1)	55.5 (51.2, 59.9)	39.9 (37.8, 41.9)	47.1 (44.2, 49.9)	46.7 (44.4, 49.1)	<0.001
2015-2016	46.6 (44.6, 48.6)	57.1 (52.8, 61.4)	38.8 (34.8, 42.8)	46.1 (43.7, 48.4)	47.0 (44.6, 49.5)	<0.001
2017-2020	46.8 (44.5, 49.2)	59.5 (55.7, 63.4)	39.1 (36.6, 41.6)	46.9 (44.0, 49.7)	47.0 (43.8, 50.2)	<0.001
**Activity**						
Total	50.7 (49.1, 52.2)	52.3 (49.3, 55.2)	45.5 (43.5, 47.5)	43.7 (42.2, 45.2)	53.2 (51.1, 55.3)	<0.001
2011-2012	50.9 (46.8, 55.0)	51.7 (46.9, 56.5)	44.8 (40.4, 49.1)	40.8 (37.2, 44.4)	54.2 (48.6, 59.7)	0.003
2013-2014	48.0 (45.3, 50.7)	53.8 (48.6, 58.9)	44.5 (40.5, 48.5)	44.3 (42.3, 46.3)	49.1 (45.8, 52.3)	0.006
2015-2016	52.2 (48.5, 55.8)	47.7 (43.1, 52.4)	47.7 (42.3, 53.0)	43.5 (40.3, 46.8)	55.5 (51.9, 59.2)	<0.001
2017-2020	51.2 (48.7, 53.8)	54.4 (48.9, 59.9)	45.3 (42.4, 48.2)	45.0 (42.6, 47.5)	53.8 (50.1, 57.4)	<0.001
**Nicotine Exposure**						
Total	70.9 (69.8, 72.0)	84.8 (83.1, 86.5)	69.0 (67.0, 70.9)	75.3 (74.1, 76.5)	69.6 (68.1, 71.1)	<0.001
2011-2012	72.0 (69.9, 74.1)	84.8 (82.6, 87.0)	71.9 (67.8, 75.9)	75.4 (73.0, 77.8)	70.2 (67.5, 73.0)	<0.001
2013-2014	70.2 (67.8, 72.5)	83.5 (80.8, 86.1)	65.8 (63.2, 68.4)	76.4 (74.0, 78.8)	68.3 (64.5, 72.2)	<0.001
2015-2016	71.0 (68.7, 73.3)	84.3 (80.3, 88.3)	68.1 (65.2, 71.0)	74.3 (72.4, 76.2)	69.5 (66.4, 72.6)	<0.001
2017-2020	71.9 (69.8, 74.1)	85.9 (82.5, 89.3)	69.7 (65.6, 73.8)	75.2 (72.9, 77.5)	70.1 (67.4, 72.8)	<0.001
**Sleep Health**						
Total	84.3 (83.7, 85.0)	86.4 (85.4, 87.5)	75.9 (74.9, 76.9)	82.7 (81.9, 83.6)	86.3 (85.5, 87.1)	<0.001
2011-2012	82.6 (81.1, 84.1)	85.0 (83.0, 87.0)	75.0 (72.6, 77.4)	82.5 (80.6, 84.4)	83.7 (81.9, 85.6)	<0.001
2013-2014	83.1 (82.0, 84.3)	86.2 (84.5, 87.9)	74.3 (72.2, 76.4)	82.0 (80.1, 83.8)	84.8 (83.5, 86.0)	<0.001
2015-2016	86.7 (85.7, 87.7)	87.0 (84.5, 89.5)	78.0 (76.2, 79.8)	83.6 (82.3, 85.0)	89.0 (88.0, 89.9)	<0.001
2017-2020	85.0 (83.8, 86.3)	87.0 (85.1, 88.9)	76.2 (74.6, 77.8)	82.8 (81.3, 84.2)	87.1 (85.5, 88.7)	<0.001
**Body Mass Index**						
Total	58.2 (57.3, 59.2)	59.4 (57.7, 61.1)	52.1 (50.9, 53.4)	54.6 (53.4, 55.8)	60.2 (58.8, 61.5)	<0.001
2011-2012	61.4 (59.3, 63.6)	65.0 (61.1, 68.9)	52.6 (50.4, 54.7)	57.2 (54.4, 59.9)	63.6 (60.8, 66.5)	<0.001
2013-2014	58.9 (57.3, 60.5)	63.5 (60.7, 66.3)	51.8 (49.1, 54.5)	56.4 (53.2, 59.5)	60.4 (58.7, 62.1)	<0.001
2015-2016	58.1 (55.8, 60.4)	61.1 (59.1, 63.1)	53.7 (50.6, 56.7)	52.8 (50.0, 55.5)	59.9 (57.3, 62.5)	0.002
2017-2020	56.0 (54.3, 57.8)	53.4 (51.3, 55.5)	51.2 (49.3, 53.1)	53.3 (51.7, 54.9)	57.9 (55.2, 60.7)	0.008
**Blood Glucose**						

© 2025 Yang H et al. *JAMA Network Open*.

Total	85.2 (84.6, 85.8)	82.5 (80.9, 84.2)	79.1 (78.1, 80.0)	82.7 (81.6, 83.7)	87.3 (86.5, 88.0)	<0.001
2011-2012	85.9 (84.8, 87.0)	84.2 (80.9, 87.6)	78.3 (75.8, 80.8)	83.6 (81.8, 85.5)	87.8 (86.1, 89.5)	<0.001
2013-2014	86.4 (85.7, 87.2)	83.4 (80.4, 86.3)	80.5 (78.9, 82.1)	84.8 (83.4, 86.1)	88.1 (87.2, 89.0)	<0.001
2015-2016	84.3 (82.6, 86.1)	83.8 (80.8, 86.8)	78.7 (76.5, 80.8)	79.9 (76.5, 83.3)	86.5 (84.8, 88.2)	<0.001
2017-2020	84.8 (83.8, 85.8)	80.4 (77.6, 83.2)	78.9 (77.2, 80.5)	82.6 (81.3, 83.9)	86.9 (85.4, 88.5)	<0.001
**Blood Lipids**						
Total	67.4 (66.5, 68.3)	67.5 (66.0, 69.0)	73.6 (72.5, 74.7)	66.6 (65.3, 67.9)	66.5 (65.4, 67.7)	<0.001
2011-2012	63.7 (61.8, 65.6)	67.9 (65.1, 70.7)	69.1 (67.2, 71.0)	63.6 (60.9, 66.4)	62.5 (60.3, 64.8)	<0.001
2013-2014	67.5 (65.9, 69.1)	67.9 (65.0, 70.7)	73.7 (71.3, 76.2)	65.5 (62.9, 68.0)	66.9 (64.9, 68.8)	0.003
2015-2016	67.6 (65.9, 69.2)	67.8 (65.1, 70.6)	74.1 (71.9, 76.2)	66.4 (64.7, 68.0)	66.7 (64.8, 68.6)	<0.001
2017-2020	69.5 (67.9, 71.1)	66.9 (64.0, 69.8)	75.9 (73.8, 78.0)	68.9 (66.5, 71.4)	68.8 (66.5, 71.0)	<0.001
**Blood Pressure**						
Total	70.7 (70.0, 71.5)	73.6 (72.3, 74.9)	63.2 (62.1, 64.3)	74.7 (73.6, 75.8)	70.8 (69.7, 71.9)	<0.001
2011-2012	71.0 (69.0, 73.0)	76.2 (73.5, 78.9)	64.3 (61.3, 67.4)	76.6 (73.4, 79.7)	70.5 (68.0, 73.1)	<0.001
2013-2014	71.4 (70.1, 72.7)	74.8 (71.8, 77.8)	63.1 (61.0, 65.2)	76.7 (74.8, 78.7)	71.4 (69.4, 73.3)	<0.001
2015-2016	69.4 (68.0, 70.7)	73.8 (70.6, 77.0)	63.9 (61.6, 66.1)	72.1 (70.0, 74.3)	69.2 (67.6, 70.9)	<0.001
2017-2020	71.0 (69.5, 72.4)	71.3 (69.5, 73.1)	62.1 (60.2, 64.0)	74.1 (72.2, 75.9)	71.8 (69.5, 74.1)	<0.001

© 2025 Yang H et al. *JAMA Network Open*.

eTable 5. Z-Scores for Racial Differences in Life's Essential 8 Components.

	Diet	Physical Activity	Nicotine Exposure	Sleep Health	Body Mass Index	Blood Glucose	Blood Lipids	Blood Pressure
**2011-2012**								
Asian-White	1.09	-0.14	2.25	0.39	0.40	-0.30	0.96	1.00
Black-White	-1.18	-1.11	0.43	-1.02	-1.35	-1.13	1.13	-0.66
Hispanic-White	-0.38	-1.67	0.93	0.03	-0.70	-0.38	0.36	1.06
**2017-2020**								
Asian-White	2.12	0.37	2.59	0.25	-0.39	-0.68	-0.01	0.20
Black-White	-0.88	-0.97	0.21	-1.33	-0.72	-0.91	1.32	-1.15
Hispanic-White	0.26	-1.01	1.01	-0.37	-0.41	-0.37	0.29	0.60

The Z-scores are calculated by standardizing values of all components across all racial and ethnicity groups.

© 2025 Yang H et al. *JAMA Network Open*.

eTable 6. Subgroup Analysis of Changes in Life's Essential 8 Score by Race and Ethnicity According to Sex and Age.

	Asian		Black		Hispanic		White
**Sex**	**Mean (95% CI)**	***P* value**	**Mean (95% CI)**	***P* value**	**Mean (95% CI)**	***P* value**	
**Male**							
Difference with White,[a] 2011-2012	2.40 (-0.96, 5.76)	0.16	-5.15 (-7.78, -2.53)	<0.001	-2.42 (-5.54, 0.70)	0.13	*Ref.*
Difference with White, 2017-2020	1.61 (-0.78, 3.99)	0.19	-3.53 (-5.76, -1.29)	0.002	-2.98 (-5.12, -0.85)	0.006	*Ref.*
dDoD[b]	-0.79 (-4.18, 2.60)	0.64	1.63 (-2.02, 5.28)	0.37	-0.56 (-3.70, 2.58)	0.72	*NA*
**Female**							
Difference with White,[a] 2011-2012	5.14 (1.43, 8.84)	0.007	-6.85 (-10.28, -3.43)	<0.001	-1.82 (-5.11, 1.46)	0.28	*Ref.*
Difference with White, 2017-2020	4.11 (1.38, 6.84)	0.003	-7.87 (-10.39, -5.34)	<0.001	-0.22 (-2.61, 2.17)	0.86	*Ref.*
dDoD[b]	-1.03 (-5.06, 3.00)	0.61	-1.01 (-5.40, 3.38)	0.65	1.61 (-2.02, 5.23)	0.38	*NA*
**Age**							
**20-40 years**							
Difference with White,[a] 2011-2012	3.59 (0.17, 7.00)	0.04	-7.30 (-10.42, -4.18)	<0.001	-3.83 (-6.82, -0.85)	0.01	*Ref.*
Difference with White, 2017-2020	3.94 (0.15, 7.73)	0.04	-3.85 (-7.01, -0.69)	0.02	-1.71 (-4.71, 1.28)	0.26	*Ref.*
dDoD[b]	0.35 (-3.93, 4.64)	0.87	3.45 (-0.69, 7.59)	0.10	2.12 (-1.59, 5.83)	0.26	*NA*
**41-60 years**							
Difference with White,[a] 2011-2012	1.81 (-1.97, 5.58)	0.35	-5.29 (-8.87, -1.71)	0.004	-3.05 (-6.49, 0.39)	0.08	*Ref.*
Difference with White, 2017-2020	0.60 (-1.71, 2.90)	0.61	-8.70 (-11.02, -6.38)	<0.001	-3.11 (-5.29, -0.93)	0.005	*Ref.*
dDoD[b]	-1.21 (-5.70, 3.29)	0.59	-3.41 (-7.92, 1.11)	0.14	-0.06 (-3.43, 3.32)	0.97	*NA*
**> 60 years**							
Difference with White,[a] 2011-2012	-0.67 (-5.01, 3.66)	0.76	-7.72 (-11.5, -3.95)	<0.001	-8.91 (-12.57, -5.26)	<0.001	*Ref.*
Difference with White, 2017-2020	0.34 (-2.25, 2.94)	0.79	-4.54 (-7.07, -2.02)	<0.001	-7.35 (-9.80, -4.90)	<0.001	*Ref.*
dDoD[b]	1.02 (-3.71, 5.75)	0.67	1.56 (-3.50, 6.63)	0.54	3.18 (-1.36, 7.72)	0.17	*NA*

a, The difference in Life's Essential 8 (LE8) score with White individuals was calculated by subtracting the LE8 values of White adults from those of Black, Latino/Hispanic, and Asian adults for both the 2011-2012 and 2017-2020 survey cycles; b, dDoD, descriptive difference-of-differences, was calculated using survey-weighted regression models that included an interaction term between survey year and race/ethnicity group.

© 2025 Yang H et al. *JAMA Network Open*.

eTable 7. Subgroup Analysis of Changes in Diet Score by Race and Ethnicity According to Sex and Age.

	Asian		Black		Hispanic		White
**Sex**	**Mean (95% CI)**	***P* value**	**Mean (95% CI)**	***P* value**	**Mean (95% CI)**	***P* value**	
**Male**							
Difference with White,[a] 2011-2012	8.32 (3.21, 13.43)	0.001	-9.31 (-14.75, -3.88)	0.001	-5.16 (-10.37, 0.06)	0.05	*Ref.*
Difference with White, 2017-2020	14.68 (7.83, 21.54)	<0.001	-6.73 (-11.58, -1.87)	0.007	-1.91 (-6.67, 2.85)	0.43	*Ref.*
DDoD[b]	6.36 (-2.15, 14.87)	0.14	2.59 (-5.00, 10.17)	0.50	3.25 (-4.46, 10.96)	0.40	*NA*
**Female**							
Difference with White,[a] 2011-2012	4.24 (-1.83, 10.31)	0.17	-10.65 (-17.06, -4.23)	0.001	-2.93 (-8.24, 2.38)	0.28	*Ref.*
Difference with White, 2017-2020	10.60 (5.61, 15.60)	<0.001	-9.05 (-13.73, -4.37)	<0.001	2.07 (-3.37, 7.51)	0.46	*Ref.*
dDoD[b]	6.36 (-1.07, 13.80)	0.09	1.60 (-6.32, 9.52)	0.69	5.00 (-2.71, 12.71)	0.20	*NA*
**Age**							
**20-40 years**							
Difference with White,[a] 2011-2012	5.47 (-0.27, 11.22)	0.06	-10.72 (-16.45, -4.99)	<0.001	-4.48 (-10.69, 1.73)	0.16	*Ref.*
Difference with White, 2017-2020	13.72 (5.85, 21.59)	0.001	-7.86 (-13.39, -2.33)	0.005	0.15 (-5.87, 6.17)	0.96	*Ref.*
dDoD[b]	8.25 (-0.92, 17.42)	0.08	2.86 (-4.97, 10.69)	0.47	4.63 (-3.50, 12.76)	0.26	*NA*
**41-60 years**							
Difference with White,[a] 2011-2012	9.35 (2.64, 16.06)	0.006	-6.93 (-13.01, -0.85)	0.03	1.51 (-5.05, 8.06)	0.65	*Ref.*
Difference with White, 2017-2020	15.40 (9.80, 21.01)	<0.001	-5.74 (-10.98, -0.51)	0.03	4.63 (-0.52, 9.78)	0.08	*Ref.*
dDoD[b]	6.06 (-2.96, 15.07)	0.18	1.18 (-6.65, 9.02)	0.76	3.12 (-5.19, 11.43)	0.46	*NA*
**> 60 years**							
Difference with White,[a] 2011-2012	7.78 (-1.05, 16.62)	0.08	-4.55 (-13.23, 4.14)	0.31	-9.53 (-17.41, -1.65)	0.02	*Ref.*
Difference with White, 2017-2020	11.13 (4.45, 17.81)	0.001	0.08 (-5.28, 5.44)	0.98	-7.19 (-12.32, -2.06)	0.006	*Ref.*
dDoD[b]	3.35 (-7.07, 13.77)	0.52	2.34 (-8.90, 13.57)	0.68	4.63 (-5.99, 15.25)	0.39	*NA*

a, The difference in Life's Essential 8 (LE8) score with White individuals was calculated by subtracting the LE8 values of White adults from those of Black, Latino/Hispanic, and Asian adults for both the 2011-2012 and 2017-2020 survey cycles; b, dDoD, descriptive difference-of-differences, was calculated using survey-weighted regression models that included an interaction term between survey year and race/ethnicity group.

© 2025 Yang H et al. *JAMA Network Open*.

eTable 8. Subgroup Analysis of Changes in Activity Score by Race and Ethnicity According to Sex and Age.

	Asian		Black		Hispanic		White
**Sex**	**Mean (95% CI)**	***P* value**	**Mean (95% CI)**	***P* value**	**Mean (95% CI)**	***P* value**	
**Male**							
Difference with White,[a] 2011-2012	1.14 (-7.37, 9.65)	0.79	-3.19 (-10.57, 4.19)	0.40	-10.02 (-17.27, -2.77)	0.007	*Ref.*
Difference with White, 2017-2020	6.16 (-2.45, 14.77)	0.16	-2.10 (-8.13, 3.94)	0.50	-7.43 (-13.3, -1.56)	0.01	*Ref.*
dDoD[b]	5.02 (-5.69, 15.73)	0.35	1.09 (-8.48, 10.66)	0.82	2.59 (-5.74, 10.92)	0.54	*NA*
**Female**							
Difference with White,[a] 2011-2012	-5.52 (-14.38, 3.34)	0.22	-14.51 (-23.92, -5.11)	0.002	-16.75 (-24.91, -8.58)	<0.001	*Ref.*
Difference with White, 2017-2020	-3.91 (-10.65, 2.82)	0.26	-13.82 (-19.11, -8.52)	<0.001	-10.11 (-14.89, -5.33)	<0.001	*Ref.*
dDoD[b]	1.60 (-8.55, 11.76)	0.75	0.69 (-10.04, 11.43)	0.90	6.64 (-2.82, 16.10)	0.17	*NA*
**Age**							
**20-40 years**							
Difference with White,[a] 2011-2012	-4.85 (-12.87, 3.18)	0.24	-11.56 (-19.58, -3.53)	0.005	-17.37 (-24.26, -10.47)	<0.001	*Ref.*
Difference with White, 2017-2020	1.05 (-7.13, 9.23)	0.80	-9.60 (-15.74, -3.47)	0.002	-9.39 (-15.45, -3.33)	0.002	*Ref.*
dDoD[b]	5.90 (-3.40, 15.19)	0.21	1.95 (-7.93, 11.84)	0.69	7.98 (-0.75, 16.70)	0.07	*NA*
**41-60 years**							
Difference with White,[a] 2011-2012	-2.76 (-11.55, 6.03)	0.54	-9.24 (-18.44, -0.05)	0.05	-16.69 (-25.28, -8.10)	<0.001	*Ref.*
Difference with White, 2017-2020	-2.15 (-11.71, 7.41)	0.66	-10.51 (-17.58, -3.44)	0.004	-12.89 (-19.64, -6.14)	<0.001	*Ref.*
dDoD[b]	0.61 (-11.18, 12.40)	0.92	-1.26 (-12.99, 10.47)	0.83	3.80 (-5.95, 13.56)	0.44	*NA*
**> 60 years**							
Difference with White,[a] 2011-2012	-7.72 (-19.84, 4.39)	0.21	-16.23 (-26.36, -6.10)	0.002	-13.31 (-22.88, -3.74)	0.006	*Ref.*
Difference with White, 2017-2020	-2.29 (-10.02, 5.44)	0.56	-11.90 (-18.86, -4.95)	0.001	-8.68 (-15.62, -1.74)	0.01	*Ref.*
dDoD[b]	5.44 (-7.89, 18.76)	0.42	4.63 (-6.99, 16.25)	0.43	4.32 (-8.36, 17.00)	0.50	*NA*

a, The difference in Life's Essential 8 (LE8) score with White individuals was calculated by subtracting the LE8 values of White adults from those of Black, Latino/Hispanic, and Asian adults for both the 2011-2012 and 2017-2020 survey cycles; b, dDoD, descriptive difference-of-differences, was calculated using survey-weighted regression models that included an interaction term between survey year and race/ethnicity group.

© 2025 Yang H et al. *JAMA Network Open*.

eTable 9. Subgroup Analysis of Changes in Nicotine Exposure Score by Race and Ethnicity According to Sex and Age.

	Asian		Black		Hispanic		White
**Sex**	**Mean (95% CI)**	***P* value**	**Mean (95% CI)**	***P* value**	**Mean (95% CI)**	***P* value**	
**Male**							
Difference with White,[a] 2011-2012	7.30 (2.31, 12.30)	0.004	-3.64 (-10.07, 2.78)	0.27	-0.62 (-5.67, 4.43)	0.81	*Ref.*
Difference with White, 2017-2020	10.11 (4.18, 16.04)	0.001	-5.54 (-10.97, -0.12)	0.04	1.29 (-3.22, 5.80)	0.58	*Ref.*
dDoD[b]	2.81 (-4.99, 10.61)	0.47	-1.90 (-9.33, 5.52)	0.61	1.91 (-4.41, 8.22)	0.55	*NA*
**Female**							
Difference with White,[a] 2011-2012	20.67 (16.49, 24.85)	<0.001	5.63 (0.47, 10.79)	0.03	11.27 (6.52, 16.01)	<0.001	*Ref.*
Difference with White, 2017-2020	20.50 (16.43, 24.57)	<0.001	3.68 (-1.66, 9.02)	0.18	9.04 (4.82, 13.27)	<0.001	*Ref.*
dDoD[b]	-0.17 (-6.42, 6.09)	0.96	-1.94 (-9.44, 5.55)	0.61	-2.22 (-9.20, 4.75)	0.53	*NA*
**Age**							
**20-40 years**							
Difference with White,[a] 2011-2012	15.02 (7.98, 22.05)	<0.001	4.91 (-4.25, 14.07)	0.29	7.47 (0.48, 14.46)	0.04	*Ref.*
Difference with White, 2017-2020	22.84 (14.77, 30.92)	<0.001	7.60 (-0.20, 15.40)	0.06	8.98 (1.91, 16.06)	0.01	*Ref.*
dDoD[b]	7.83 (-3.69, 19.35)	0.18	2.69 (-8.20, 13.59)	0.62	1.51 (-8.75, 11.77)	0.77	*NA*
**41-60 years**							
Difference with White,[a] 2011-2012	17.75 (12.18, 23.33)	<0.001	2.71 (-4.43, 9.86)	0.46	8.41 (3.61, 13.22)	0.001	*Ref.*
Difference with White, 2017-2020	14.66 (10.04, 19.29)	<0.001	-3.81 (-10.02, 2.40)	0.23	5.65 (0.48, 10.82)	0.03	*Ref.*
dDoD[b]	-3.09 (-9.31, 3.13)	0.32	-6.53 (-15.31, 2.25)	0.14	-2.77 (-10.01, 4.48)	0.45	*NA*
**> 60 years**							
Difference with White,[a] 2011-2012	12.05 (6.84, 17.26)	<0.001	-2.08 (-7.93, 3.77)	0.49	-4.46 (-10.84, 1.92)	0.17	*Ref.*
Difference with White, 2017-2020	5.80 (1.65, 9.95)	0.006	1.86 (-2.29, 6.01)	0.38	-7.83 (-12.88, -2.78)	0.002	*Ref.*
dDoD[b]	-6.25 (-12.82, 0.32)	0.06	-3.37 (-11.22, 4.47)	0.39	3.94 (-3.94, 11.82)	0.32	*NA*

a, The difference in Life's Essential 8 (LE8) score with White individuals was calculated by subtracting the LE8 values of White adults from those of Black, Latino/Hispanic, and Asian adults for both the 2011-2012 and 2017-2020 survey cycles; b, dDoD, descriptive difference-of-differences, was calculated using survey-weighted regression models that included an interaction term between survey year and race/ethnicity group.

© 2025 Yang H et al. *JAMA Network Open*.

eTable 10. Subgroup Analysis of Changes in Sleep Health Score by Race and Ethnicity According to Sex and Age.

	Asian		Black		Hispanic		White
**Sex**	**Mean (95% CI)**	***P* value**	**Mean (95% CI)**	***P* value**	**Mean (95% CI)**	***P* value**	
**Male**							
Difference with White,[a] 2011-2012	1.53 (-1.02, 4.08)	0.24	-10.03 (-12.57, -7.49)	<0.001	-0.46 (-3.67, 2.75)	0.78	*Ref.*
Difference with White, 2017-2020	1.21 (-2.61, 5.02)	0.54	-10.77 (-13.54, -8.00)	<0.001	-4.22 (-7.23, -1.21)	0.006	*Ref.*
dDoD[b]	-0.32 (-4.32, 3.67)	0.87	-0.74 (-4.32, 2.84)	0.68	-3.76 (-7.72, 0.20)	0.06	*NA*
**Female**							
Difference with White,[a] 2011-2012	1.04 (-2.46, 4.53)	0.56	-7.69 (-11.73, -3.66)	<0.001	-2.03 (-5.06, 1.01)	0.19	*Ref.*
Difference with White, 2017-2020	-1.37 (-3.88, 1.14)	0.28	-11.15 (-13.73, -8.57)	<0.001	-4.48 (-6.75, -2.22)	<0.001	*Ref.*
dDoD[b]	-2.41 (-6.62, 1.80)	0.26	-3.45 (-7.73, 0.83)	0.11	-2.46 (-5.75, 0.83)	0.14	*NA*
**Age**							
**20-40 years**							
Difference with White,[a] 2011-2012	5.95 (2.35, 9.55)	0.001	-6.12 (-10.23, -2.00)	0.004	1.69 (-1.83, 5.21)	0.35	*Ref.*
Difference with White, 2017-2020	1.34 (-2.05, 4.74)	0.44	-10.81 (-13.81, -7.82)	<0.001	-4.44 (-7.23, -1.65)	0.002	*Ref.*
dDoD[b]	-4.61 (-8.49, -0.73)	0.02	-4.70 (-9.31, -0.08)	0.04	-6.13 (-9.74, -2.52)	0.001	*NA*
**41-60 years**							
Difference with White,[a] 2011-2012	-0.19 (-4.37, 3.99)	0.93	-9.73 (-13.95, -5.50)	<0.001	-2.22 (-6.35, 1.90)	0.29	*Ref.*
Difference with White, 2017-2020	0.17 (-3.14, 3.47)	0.92	-11.64 (-15.53, -7.75)	<0.001	-3.87 (-7.62, -0.12)	0.04	*Ref.*
dDoD[b]	0.36 (-4.58, 5.29)	0.89	-1.92 (-6.89, 3.05)	0.44	-1.64 (-6.85, 3.57)	0.53	*NA*
**> 60 years**							
Difference with White,[a] 2011-2012	-6.35 (-10.92, -1.79)	0.006	-6.99 (-10.58, -3.40)	<0.001	-11.15 (-14.38, -7.92)	<0.001	*Ref.*
Difference with White, 2017-2020	-4.50 (-8.76, -0.24)	0.04	-5.11 (-8.49, -1.73)	0.003	-9.63 (-13.24, -6.02)	<0.001	*Ref.*
dDoD[b]	1.86 (-4.58, 8.29)	0.57	1.52 (-2.70, 5.75)	0.47	1.88 (-2.83, 6.58)	0.43	*NA*

a, The difference in Life's Essential 8 (LE8) score with White individuals was calculated by subtracting the LE8 values of White adults from those of Black, Latino/Hispanic, and Asian adults for both the 2011-2012 and 2017-2020 survey cycles; b, dDoD, descriptive difference-of-differences, was calculated using survey-weighted regression models that included an interaction term between survey year and race/ethnicity group.

© 2025 Yang H et al. *JAMA Network Open*.

eTable 11. Subgroup Analysis of Changes in Body Mass Index Score by Race and Ethnicity According to Sex and Age.

	Asian		Black		Hispanic		White
**Sex**	**Mean (95% CI)**	***P* value**	**Mean (95% CI)**	***P* value**	**Mean (95% CI)**	***P* value**	
**Male**							
Difference with White,[a] 2011-2012	-1.83 (-7.55, 3.89)	0.53	-2.34 (-6.05, 1.37)	0.22	-5.45 (-9.91, -0.98)	0.02	*Ref.*
Difference with White, 2017-2020	-10.29 (-14.86, -5.72)	<0.001	2.36 (-2.96, 7.68)	0.39	-4.12 (-8.44, 0.19)	0.06	*Ref.*
dDoD[b]	-8.46 (-15.78, -1.13)	0.02	4.69 (-1.40, 10.79)	0.13	1.33 (-4.34, 6.99)	0.64	*NA*
**Female**							
Difference with White,[a] 2011-2012	4.17 (-1.38, 9.71)	0.14	-18.47 (-23.03, -13.92)	<0.001	-7.49 (-12.19, -2.80)	0.002	*Ref.*
Difference with White, 2017-2020	0.14 (-5.12, 5.39)	0.96	-14.62 (-19.49, -9.75)	<0.001	-5.03 (-9.44, -0.62)	0.03	*Ref.*
dDoD[b]	-4.03 (-11.34, 3.28)	0.27	3.85 (-2.47, 10.17)	0.23	2.46 (-3.80, 8.73)	0.44	*NA*
**Age**							
**20-40 years**							
Difference with White,[a] 2011-2012	1.98 (-4.57, 8.53)	0.55	-14.85 (-19.47, -10.24)	<0.001	-8.68 (-14.36, -2.99)	0.003	*Ref.*
Difference with White, 2017-2020	-6.62 (-13.20, -0.03)	0.05	-3.17 (-9.60, 3.25)	0.33	-4.46 (-10.25, 1.32)	0.13	*Ref.*
dDoD[b]	-8.60 (-16.91, -0.28)	0.04	11.68 (3.20, 20.15)	0.008	4.21 (-2.77, 11.20)	0.23	*NA*
**41-60 years**							
Difference with White,[a] 2011-2012	-0.22 (-4.63, 4.18)	0.92	-8.89 (-13.63, -4.14)	<0.001	-6.78 (-11.36, -2.19)	0.004	*Ref.*
Difference with White, 2017-2020	-4.66 (-10.84, 1.53)	0.14	-12.89 (-17.19, -8.59)	<0.001	-6.17 (-11.03, -1.30)	0.01	*Ref.*
dDoD[b]	-4.43 (-12.07, 3.20)	0.25	-4.00 (-10.87, 2.87)	0.25	0.61 (-4.96, 6.18)	0.83	*NA*
**> 60 years**							
Difference with White,[a] 2011-2012	-2.59 (-9.05, 3.88)	0.43	-8.77 (-16.74, -0.81)	0.03	-9.74 (-17.25, -2.24)	0.01	*Ref.*
Difference with White, 2017-2020	-0.81 (-7.80, 6.18)	0.82	-5.45 (-9.89, -1.01)	0.02	-4.51 (-9.17, 0.15)	0.06	*Ref.*
dDoD[b]	1.77 (-8.00, 11.55)	0.72	5.23 (-3.39, 13.85)	0.23	3.32 (-5.65, 12.30)	0.46	*NA*

a, The difference in Life's Essential 8 (LE8) score with White individuals was calculated by subtracting the LE8 values of White adults from those of Black, Latino/Hispanic, and Asian adults for both the 2011-2012 and 2017-2020 survey cycles; b, dDoD, descriptive difference-of-differences, was calculated using survey-weighted regression models that included an interaction term between survey year and race/ethnicity group.

© 2025 Yang H et al. *JAMA Network Open*.

eTable 12. Subgroup Analysis of Changes in Blood Glucose Score by Race and Ethnicity According to Sex and Age.

	Asian		Black		Hispanic		White
**Sex**	**Mean (95% CI)**	***P* value**	**Mean (95% CI)**	***P* value**	**Mean (95% CI)**	***P* value**	
**Male**							
Difference with White,[a] 2011-2012	-4.37 (-8.89, 0.14)	0.06	-10.73 (-14.15, -7.32)	<0.001	-3.87 (-7.01, -0.73)	0.02	*Ref.*
Difference with White, 2017-2020	-7.28 (-11.48, -3.08)	0.001	-7.06 (-10.74, -3.39)	<0.001	-4.21 (-7.44, -0.99)	0.01	*Ref.*
dDoD[b]	-2.90 (-8.39, 2.58)	0.29	3.67 (-1.33, 8.67)	0.15	-0.35 (-4.37, 3.68)	0.86	*NA*
**Female**							
Difference with White,[a] 2011-2012	-2.87 (-7.23, 1.50)	0.20	-8.48 (-12.57, -4.39)	<0.001	-4.42 (-8.02, -0.81)	0.02	*Ref.*
Difference with White, 2017-2020	-5.92 (-9.78, -2.06)	0.003	-9.01 (-11.29, -6.72)	<0.001	-4.48 (-7.27, -1.69)	0.002	*Ref.*
dDoD[b]	-3.06 (-9.10, 2.98)	0.32	-0.53 (-5.69, 4.64)	0.84	-0.06 (-4.86, 4.73)	0.98	*NA*
**Age**							
**20-40 years**							
Difference with White,[a] 2011-2012	-2.89 (-5.51, -0.27)	0.03	-8.28 (-11.31, -5.25)	<0.001	-3.74 (-6.51, -0.96)	0.008	*Ref.*
Difference with White, 2017-2020	-3.79 (-6.63, -0.96)	0.009	-5.42 (-7.31, -3.53)	<0.001	-4.06 (-6.04, -2.08)	<0.001	*Ref.*
dDoD[b]	-0.90 (-4.62, 2.81)	0.63	2.86 (-0.98, 6.71)	0.14	-0.32 (-3.96, 3.31)	0.86	*NA*
**41-60 years**							
Difference with White,[a] 2011-2012	-9.44 (-13.93, -4.94)	<0.001	-13.06 (-17.44, -8.69)	<0.001	-11.96 (-16.25, -7.68)	<0.001	*Ref.*
Difference with White, 2017-2020	-9.04 (-13.59, -4.49)	<0.001	-12.97 (-16.87, -9.06)	<0.001	-8.14 (-12.45, -3.83)	<0.001	*Ref.*
dDoD[b]	0.39 (-6.41, 7.19)	0.91	0.10 (-6.25, 6.44)	0.98	3.82 (-2.15, 9.79)	0.21	*NA*
**> 60 years**							
Difference with White,[a] 2011-2012	-5.96 (-13.14, 1.22)	0.10	-13.1 (-18.73, -7.47)	<0.001	-14.92 (-19.19, -10.66)	<0.001	*Ref.*
Difference with White, 2017-2020	-15.7 (-23.71, -7.69)	<0.001	-14.76 (-19.21, -10.31)	<0.001	-12.48 (-16.31, -8.66)	<0.001	*Ref.*
dDoD[b]	-9.74 (-21.17, 1.70)	0.09	2.44 (-4.25, 9.13)	0.47	-1.66 (-9.45, 6.14)	0.67	*NA*

a, The difference in Life's Essential 8 (LE8) score with White individuals was calculated by subtracting the LE8 values of White adults from those of Black, Latino/Hispanic, and Asian adults for both the 2011-2012 and 2017-2020 survey cycles; b, dDoD, descriptive difference-of-differences, was calculated using survey-weighted regression models that included an interaction term between survey year and race/ethnicity group.

© 2025 Yang H et al. *JAMA Network Open*.

eTable 13. Subgroup Analysis of Changes in Blood Lipids Score by Race and Ethnicity According to Sex and Age.

	Asian		Black		Hispanic		White
**Sex**	**Mean (95% CI)**	***P* value**	**Mean (95% CI)**	***P* value**	**Mean (95% CI)**	***P* value**	
**Male**							
Difference with White,[a] 2011-2012	2.01 (-2.61, 6.63)	0.39	7.48 (3.70, 11.26)	<0.001	-1.98 (-6.34, 2.37)	0.37	*Ref.*
Difference with White, 2017-2020	-7.38 (-12.01, -2.76)	0.002	8.27 (4.38, 12.16)	<0.001	-2.54 (-7.10, 2.01)	0.27	*Ref.*
dDoD[b]	-9.39 (-15.34, -3.44)	0.003	0.79 (-4.51, 6.10)	0.77	-0.56 (-7.54, 6.42)	0.87	*NA*
**Female**							
Difference with White,[a] 2011-2012	8.23 (4.36, 12.10)	<0.001	5.69 (2.12, 9.26)	0.002	4.27 (-0.13, 8.66)	0.06	*Ref.*
Difference with White, 2017-2020	2.54 (-1.97, 7.05)	0.27	5.97 (2.25, 9.69)	0.002	3.01 (-0.93, 6.96)	0.14	*Ref.*
dDoD[b]	-5.69 (-11.29, -0.08)	0.04	0.28 (-4.53, 5.09)	0.91	-1.26 (-6.78, 4.26)	0.65	*NA*
**Age**							
**20-40 years**							
Difference with White,[a] 2011-2012	3.10 (-1.59, 7.79)	0.19	0.97 (-3.84, 5.78)	0.69	-3.97 (-9.34, 1.41)	0.15	*Ref.*
Difference with White, 2017-2020	-4.13 (-9.67, 1.40)	0.14	5.73 (1.60, 9.86)	0.007	-1.86 (-5.98, 2.26)	0.38	*Ref.*
dDoD[b]	-7.24 (-14.59, 0.12)	0.05	4.76 (-1.34, 10.87)	0.12	2.11 (-5.26, 9.48)	0.57	*NA*
**41-60 years**							
Difference with White,[a] 2011-2012	2.90 (-1.78, 7.58)	0.23	8.72 (4.55, 12.90)	<0.001	0.68 (-4.11, 5.47)	0.78	*Ref.*
Difference with White, 2017-2020	-5.70 (-10.73, -0.66)	0.03	5.88 (1.68, 10.08)	0.006	-3.44 (-7.63, 0.74)	0.11	*Ref.*
dDoD[b]	-8.60 (-15.61, -1.59)	0.02	-2.84 (-9.07, 3.39)	0.37	-4.12 (-9.85, 1.60)	0.16	*NA*
**> 60 years**							
Difference with White,[a] 2011-2012	3.31 (-4.63, 11.25)	0.41	-4.10 (-10.26, 2.06)	0.19	6.71 (1.62, 11.80)	0.010	*Ref.*
Difference with White, 2017-2020	4.72 (-2.89, 12.33)	0.22	1.89 (-2.28, 6.07)	0.37	6.75 (1.85, 11.66)	0.007	*Ref.*
dDoD[b]	1.41 (-9.96, 12.78)	0.81	0.05 (-7.21, 7.31)	0.99	5.99 (-1.29, 13.28)	0.11	*NA*

a, The difference in Life's Essential 8 (LE8) score with White individuals was calculated by subtracting the LE8 values of White adults from those of Black, Latino/Hispanic, and Asian adults for both the 2011-2012 and 2017-2020 survey cycles; b, dDoD, descriptive difference-of-differences, was calculated using survey-weighted regression models that included an interaction term between survey year and race/ethnicity group.

© 2025 Yang H et al. *JAMA Network Open*.

eTable 14. Subgroup Analysis of Changes in Blood Pressure Score by Race and Ethnicity According to Sex and Age.

	Asian		Black		Hispanic		White
**Sex**	**Mean (95% CI)**	***P* value**	**Mean (95% CI)**	***P* value**	**Mean (95% CI)**	***P* value**	
**Male**							
Difference with White,[a] 2011-2012	3.30 (-2.22, 8.83)	0.24	-6.85 (-11.67, -2.03)	0.005	5.83 (0.55, 11.11)	0.03	*Ref.*
Difference with White, 2017-2020	-1.67 (-5.71, 2.37)	0.42	-7.93 (-12.14, -3.72)	<0.001	0.09 (-4.37, 4.55)	0.97	*Ref.*
dDoD[b]	-4.97 (-11.77, 1.82)	0.15	-1.08 (-7.78, 5.62)	0.75	-5.74 (-12.26, 0.78)	0.08	*NA*
**Female**							
Difference with White,[a] 2011-2012	7.58 (3.44, 11.72)	<0.001	-5.81 (-10.42, -1.20)	0.014	6.48 (2.3, 10.67)	0.002	*Ref.*
Difference with White, 2017-2020	0.59 (-3.07, 4.24)	0.75	-11.41 (-14.27, -8.55)	<0.001	4.81 (1.04, 8.57)	0.01	*Ref.*
dDoD[b]	-6.99 (-11.51, -2.47)	0.003	-5.60 (-11.17, -0.03)	0.04	-1.68 (-7.27, 3.92)	0.55	*NA*
**Age**							
**20-40 years**							
Difference with White,[a] 2011-2012	4.72 (0.64, 8.80)	0.02	-6.94 (-12.13, -1.75)	0.009	2.64 (-1.84, 7.12)	0.25	*Ref.*
Difference with White, 2017-2020	1.71 (-3.30, 6.72)	0.50	-4.72 (-8.87, -0.56)	0.03	2.30 (-1.37, 5.97)	0.22	*Ref.*
dDoD[b]	-3.01 (-9.36, 3.35)	0.35	2.23 (-4.42, 8.88)	0.51	-0.34 (-6.09, 5.40)	0.91	*NA*
**41-60 years**							
Difference with White,[a] 2011-2012	3.27 (-0.40, 6.95)	0.08	-8.32 (-12.61, -4.03)	<0.001	1.73 (-2.58, 6.03)	0.43	*Ref.*
Difference with White, 2017-2020	-2.20 (-6.85, 2.45)	0.35	-15.68 (-19.44, -11.92)	<0.001	-2.99 (-7.18, 1.20)	0.16	*Ref.*
dDoD[b]	-5.47 (-11.31, 0.37)	0.07	-7.36 (-12.91, -1.80)	0.010	-4.71 (-11.13, 1.70)	0.15	*NA*
**> 60 years**							
Difference with White,[a] 2011-2012	-3.99 (-12.00, 4.02)	0.33	-5.64 (-13.26, 1.97)	0.15	-12.41 (-17.00, -7.81)	<0.001	*Ref.*
Difference with White, 2017-2020	-13.43 (-19.37, -7.49)	<0.001	-7.46 (-12.77, -2.16)	0.006	-19.00 (-23.95, -14.05)	<0.001	*Ref.*
dDoD[b]	-9.44 (-18.74, -0.13)	0.04	-6.59 (-14.14, 0.96)	0.09	-1.82 (-11.66, 8.02)	0.71	*NA*

a, The difference in Life's Essential 8 (LE8) score with White individuals was calculated by subtracting the LE8 values of White adults from those of Black, Latino/Hispanic, and Asian adults for both the 2011-2012 and 2017-2020 survey cycles; b, dDoD, descriptive difference-of-differences, was calculated using survey-weighted regression models that included an interaction term between survey year and race/ethnicity group.

© 2025 Yang H et al. *JAMA Network Open*.

eTable 15. Sensitivity Analysis of Changes in Life's Essential 8 Score and Its Components by Race and Ethnicity.

	Asian		Black		Hispanic		White
	**Mean (95% CI)**	***P* value**	**Mean (95% CI)**	***P* value**	**Mean (95% CI)**	***P* value**	
**LE8 Score**							
Difference with White,[a] 2011-2012	2.57 (0.57, 4.57)	0.07	-3.40 (-5.44, -1.35)	0.03	0.43 (-1.08, 1.93)	0.61	*Ref.*
Difference with White, 2017-2020	1.76 (0.23, 3.30)	0.04	-3.01 (-4.47, -1.55)	0.002	0.81 (-0.63, 2.25)	0.29	*Ref.*
dDoD[b]	-1.04 (-3.63, 1.55)	0.42	0.25 (-1.98, 2.48)	0.82	0.13 (-1.79, 2.04)	0.90	*NA*
**Diet**							
Difference with White, 2011-2012	7.13 (2.28, 11.97)	0.04	-5.22 (-10.36, -0.09)	0.12	4.07 (0.12, 8.02)	0.11	*Ref.*
Difference with White, 2017-2020	13.85 (10.15, 17.56)	<0.001	-2.68 (-5.37, 0.01)	0.08	7.22 (3.05, 11.39)	0.005	*Ref.*
dDoD[b]	6.56 (0.29, 12.84)	0.04	2.87 (-2.64, 8.38)	0.30	3.16 (-2.29, 8.61)	0.25	*NA*
**Physical Activity**							
Difference with White, 2011-2012	-2.30 (-7.91, 3.30)	0.47	-5.69 (-10.58, -0.81)	0.08	-6.27 (-12.37, -0.17)	0.11	*Ref.*
Difference with White, 2017-2020	1.87 (-4.23, 7.97)	0.56	-3.24 (-7.95, 1.48)	0.20	-0.36 (-4.68, 3.96)	0.87	*Ref.*
dDoD[b]	3.39 (-5.13, 11.92)	0.43	0.64 (-5.76, 7.04)	0.84	2.98 (-4.12, 10.08)	0.40	*NA*
**Nicotine Exposure**							
Difference with White, 2011-2012	14.23 (10.31, 18.16)	0.002	5.17 (1.69, 8.64)	0.04	14.43 (8.50, 20.36)	0.009	*Ref.*
Difference with White, 2017-2020	15.92 (12.66, 19.19)	<0.001	8.35 (4.20, 12.49)	0.002	13.79 (10.02, 17.57)	<0.001	*Ref.*
dDoD[b]	1.04 (-3.68, 5.76)	0.66	0.59 (-4.88, 6.07)	0.83	-2.45 (-8.98, 4.08)	0.45	*NA*
**Sleep Health**							
Difference with White, 2011-2012	1.90 (-0.40, 4.19)	0.18	-6.44 (-9.72, -3.16)	0.02	1.24 (-0.77, 3.25)	0.29	*Ref.*
Difference with White, 2017-2020	0.57 (-1.77, 2.92)	0.64	-8.12 (-11.04, -5.20)	<0.001	-0.95 (-3.62, 1.73)	0.50	*Ref.*
dDoD[b]	-1.02 (-4.43, 2.38)	0.55	-2.25 (-6.10, 1.60)	0.25	-2.44 (-5.32, 0.44)	0.09	*NA*
**Body Mass Index**							
Difference with White, 2011-2012	0.25 (-5.48, 5.97)	0.94	-8.42 (-13.09, -3.74)	0.02	-6.46 (-10.22, -2.69)	0.03	*Ref.*
Difference with White, 2017-2020	-3.87 (-7.63, -0.10)	0.07	-6.20 (-10.22, -2.18)	0.01	-5.35 (-8.81, -1.89)	0.010	*Ref.*
dDoD[b]	-4.45 (-11.47, 2.57)	0.21	3.85 (-1.65, 9.35)	0.17	2.21 (-2.98, 7.41)	0.40	*NA*
**Blood Glucose**							
Difference with White, 2011-2012	-4.02 (-6.71, -1.34)	0.04	-7.29 (-9.82, -4.75)	0.005	-3.59 (-5.31, -1.88)	0.02	*Ref.*
Difference with White, 2017-2020	-6.13 (-9.06, -3.19)	0.002	-7.42 (-9.42, -5.42)	<0.001	-4.48 (-6.41, -2.55)	0.001	*Ref.*
dDoD[b]	-2.01 (-6.22, 2.20)	0.34	0.31 (-2.75, 3.35)	0.84	0.36 (-2.11, 2.82)	0.77	*NA*
**Blood Lipids**							
Difference with White, 2011-2012	3.05 (-0.22, 6.32)	0.14	4.87 (1.65, 8.09)	0.04	-1.66 (-5.10, 1.78)	0.40	*Ref.*
Difference with White, 2017-2020	-3.83 (-7.56, -0.10)	0.07	3.94 (1.21, 6.67)	0.02	-2.77 (-6.32, 0.78)	0.15	*Ref.*
dDoD[b]	-7.42 (-12.41, -2.43)	0.004	0.25 (-3.90, 4.39)	0.91	-0.60 (-5.78, 4.58)	0.82	*NA*

© 2025 Yang H et al. *JAMA Network Open*.

**Blood Pressure**							
Difference with White, 2011-2012	0.30 (-2.80, 3.41)	0.86	-4.13 (-7.23, -1.04)	0.06	1.66 (-1.23, 4.55)	0.32	*Ref.*
Difference with White, 2017-2020	-4.29 (-6.72, -1.85)	0.005	-8.70 (-11.67, -5.74)	<0.001	-0.64 (-3.73, 2.45)	0.69	*Ref.*
dDoD[b]	-4.40 (-8.34, -0.46)	0.03	-4.25 (-8.65, 0.15)	0.06	-2.21 (-6.76, 2.35)	0.34	*NA*

a, The difference in Life's Essential 8 (LE8) score with White individuals was calculated by subtracting the LE8 values of White adults from those of Black, Latino/Hispanic, and Asian adults for both the 2011-2012 and 2017-2020 survey cycles; b, dDoD, descriptive difference-of-differences, was calculated using survey-weighted regression models that included an interaction term between survey year and race/ethnicity group. Adjusted for age, sex (male, female), family income-to-poverty ratio (≤1.30, 1.31–1.85, 1.86–3.50, and >3.50), education level (less than high school, high school graduate, and more than high school), marital status (unmarried, married or living with a partner), insurance status (uninsured, insured), medication use for hypertension, diabetes, and hyperlipidemia, and the presence of depression.

© 2025 Yang H et al. *JAMA Network Open*.
