# Supplementary material for: Trends and Key Factors Associated With Racial and Ethnic Differences in Life’s Essential 8 Scores
Source: JAMA Netw Open. 2025 Jun 18;8(6):e2516663. doi: 10.1001/jamanetworkopen.2025.16663 (PMC12177675; doi:10.1001/jamanetworkopen.2025.16663)
Supplement: Supplement 2. — Data Sharing Statement [file jamanetwopen-e2516663-s002.pdf]

Data Sharing Statement

Yang. Trends and Key Factors Associated With Racial and Ethnic Differences in Life's Essential 8 Scores. *JAMA Netw Open*. Published June 18, 2025. doi:10.1001/jamanetworkopen.2025.16663

Data
Data available: Yes
Data types: Deidentified participant data
How to access data: The data (National Health and Nutrition Examination Survey) used in this study are available publicly via the Centers for Disease Control and Prevention website: https://wwwn.cdc.gov/nchs/nhanes/Default.aspx.
When available: With publication

Supporting Documents
Document types: None

Additional Information
Who can access the data: The data used in this study are publicly available to researchers and anyone requesting access through the Centers for Disease Control and Prevention (CDC) website.
Types of analyses: The data will be made available for any purpose.
Mechanisms of data availability: The data will be made available without investigator support and do not require proposal approval or a data access agreement. They can be freely accessed through the CDC's National Health and Nutrition Examination Survey (NHANES) website.
Any additional restrictions: NA
